# Privacy Fact Sheets for Mitigating Disease-Related Privacy Concerns and Facilitating Equal Access to the Electronic Health Record: Randomized Controlled Trial

**DOI:** 10.2196/71124

**Published:** 2026-01-15

**Authors:** Niklas von Kalckreuth, Markus A Feufel

**Affiliations:** 1 Division of Ergonomics, Department of Psychology and Ergonomics, Technische Universität Berlin, Straße des 17. Juni 135, Berlin, 10623, Germany, 49 3031470747

**Keywords:** adoption, disease characteristics, EHR, electronic health record, mHealth, mobile health, stigma, time course, transparency, transparency feature, privacy concern

## Abstract

**Background:**

The German electronic health record (EHR) aims to enhance patient care and reduce costs, but users often worry about data privacy and security. To mitigate disease-related privacy concerns, for instance, surrounding stigmatized diseases, we test the effect of privacy fact sheets (PFSs)—a concise but comprehensive transparency feature designed to increase users’ perceived control over their data—on increasing EHR use in a simulated online study.

**Objective:**

The study aimed to investigate whether displaying a PFS shortly before upload decisions must be made mitigates disease-related privacy concerns and makes uploads more likely.

**Methods:**

In an online survey study, 393 German participants from the recruitment platform Prolific were asked to interact with a randomly assigned medical report that varied systematically in terms of disease-related stigma (high vs low) and time course (TC; acute vs chronic). They were then asked to decide whether to upload the report to an EHR click dummy, while we systematically varied the presentation of privacy information (PFS vs no PFS). Participants were randomly (single-blinded) assigned to one of the 2×2×2 conditions (stigma, TC, privacy information).

**Results:**

All 393 participants were randomly assigned to one of the following groups: low, acute, no PFS (n=52, 13.2%); low, chronic, no PFS (n=45, 11.5%); high, acute, no PFS (n=46, 11.7%); high, chronic, no PFS (n=55, 14%); low, acute, PFS (n=44, 11.2%); low, chronic, PFS (n=41, 10.4%); high, acute, PFS (n=56, 14.2%); and high, chronic, PFS (n=54, 13.7%). The results show that, in general, upload behavior is negatively influenced by disease-related stigma (odds ratio [OR] 0.130; *P*<.001) and positively influenced when a PFS is given (OR 4.527; *P*<.001). This increase was particularly pronounced for stigmatized diseases (OR 5.952; *P*=.006), but independent of the TC of the diseases.

**Conclusions:**

Our results demonstrate that PFSs may help to increase EHR uploads in people interacting with a realistic EHR click dummy, by mitigating privacy concerns in scenarios involving stigmatized diseases. Results further indicate that a PFS is mainly relevant and effective for people with increased privacy risk perceptions, whereas they neither benefit nor hurt others. Thus, implementing PFSs may increase the likelihood that users who perceive high privacy risks when confronted with sensitive or stigmatized health information decide to upload their data to the EHR, ultimately increasing digital health equity.

## Introduction

### Background

The electronic health record (EHR) is one key element in the digital transformation of health care systems because it allows patients’ health data (eg, diagnoses, therapies, vaccinations, and medication plans) to be readily documented, exchanged, and viewed by various stakeholders [1-6]. The resulting network of care providers can make patient treatment more effective, safer, and faster across institutions [7]. For instance, preexisting conditions, intolerances, and medication plans can be taken into account during diagnosis and treatment to prevent adverse medication interactions, duplicate diagnoses, overtreatment, and undertreatment [5]. Also, it is hoped that physicians will spend less time on obtaining patients’ medical history thanks to the EHR, which they could devote to actual patient treatment [8]. In Germany, approximately 90% of residents are covered by statutory health insurance and thus received an EHR account by default in January 2025, unless they opted out [9-11]. From October 1, 2025, health care providers are required to use the EHR infrastructure for documentation and data exchange [11]. A prerequisite for the potential of EHRs to realize is user engagement. Specifically, the Patient Data Protection Act mandates that patients maintain sole control of their data, allowing them to decide which information is stored in the EHR, who has access to it, and which data are to be deleted [5]. Consequently, the success of the EHR hinges on patients’ actual use of the technology. But national and international studies indicate that many patients remain skeptical toward the EHR, mainly due to concerns arising from limited trust in both data security (ie, the technical and organizational measures that protect personal data) and data privacy (ie, the rights and rules governing how personal data is collected, used, and shared) [8,12-15]. In Germany, these concerns were intensified due to reported security issues, which were addressed and resolved during the rollout [16,17]. However, concerns about EHR use are not static in nature. Our previous studies suggest that participants adapt their attitudes toward EHR dynamically. For instance, we could show that the perceived risks and benefits of using EHR are related to disease-specific privacy concerns, such as the stigma and the time course (TC) of diseases, that is, the more permanent and risky nature of data associated with chronic conditions [18-20]. Previous studies have also shown that existing communication strategies, for instance, by health insurers or the Federal Ministry of Health, have not been sufficient to effectively convey the core functions and data protection mechanisms of the German EHR [13,14,21]. Against this background, there is a need for concise and comprehensible communication strategies that can transparently explain data privacy and data security measures, thereby strengthening user trust in the EHR. In this study, we investigate the effect of a privacy fact sheet (PFS)—a concise but comprehensive transparency feature—on increasing EHR use and, specifically, to what extent the PFS can mitigate disease-related privacy concerns and increase the upload of medical reports to the EHR.

### Prior Work

“Notice and choice” is the most widely used framework for ensuring data privacy worldwide [22,23]. As its name suggests, it consists of 2 components: privacy notices and privacy choices. Whereas *privacy notices* explain how personal data are collected, processed, and shared with third parties, *privacy choices* give users control over various aspects of these practices, including the decision to start and terminate them [22]. Various studies indicate that, if informed by privacy notices, users are empowered to choose IT systems that match their preferences, typically those with high data security and privacy standards, and avoid less secure ones [24,25]. But the current formats used for privacy notices, most commonly *privacy policies*, tend to provide rather detailed information and often use legal jargon [26-30], which aims to maximize legal protection of IT providers rather than to transparently inform users [31]. Research has shown that overly lengthy and complex privacy policies may ultimately serve as a “red flag,” leading users to lose trust in the provider, if not to discontinue technology use altogether [32,33]. Consequently, concise, easy-to-understand privacy notices are a prerequisite for users to adopt digital health technologies such as the EHR [33-35].

In contrast to full-blown privacy policies, the shorter *transparency features* have been shown to be an effective type of privacy notice, because they provide a brief and easy-to-understand overview of data privacy and data security measures and are meant to inform rather than to provide legal assurance [36,37]. Recent work also shows that such transparency features can decrease privacy concerns and increase EHR acceptance by strengthening users’ perceived control over personal data—a construct that describes the extent to which users feel able to understand, oversee, and control how their personal data are handled and that plays a central role in privacy calculus models [7,38-40]. Stronger perceptions of control are known to reduce privacy concerns and foster trust in digital health and online technologies [7,38,39,41].

Empirical studies across domains suggest that transparency features may influence user behavior. For instance, a study in the eCommerce domain demonstrated that displaying a transparency feature positively influences purchase numbers [37]. But increased use does not (only) depend on the format of the privacy notice; it is also influenced by the contents it provides, including the efficacy of the mentioned data protection measures and privacy choices, and its timing, that is, when the privacy notice is given to users [42]. At the same time, studies in other digital contexts have reported mixed results regarding the behavioral impact of privacy notices and transparency features, suggesting that their effectiveness may depend on contextual factors such as perceived privacy risk or data sensitivity [36,43]. Our previous studies have shown that a transparency feature with a concise but comprehensive summary of all relevant contents—which we refer to as a PFS—positively influences EHR use when given shortly before the upload process [40,44]. In addition, we could show that a patient-centered framing of these contents that specifies what users can do to control the EHR and emphasizes their perceived control over personal data (eg, you can control all of your data) has the biggest effect on EHR adoption [44].

As stated at the outset, in another line of studies, we have shown that privacy concerns, intention to use the EHR, and upload behavior are influenced by the characteristics of diseases, in particular by disease-related stigma and TC [18-20]. Disease-specific stigma has been shown to have an inhibiting influence on upload behavior and to increase the risks people perceive when they are asked to upload information related to these diseases to the EHR [18,19]. Conversely, the TC of diseases (ie, whether diseases are chronic rather than acute) tends to increase both privacy concerns and intention to use the EHR. That is, patients with chronic conditions recognize a greater value in using the EHR but have heightened privacy concerns when it comes to uploading chronic conditions to the EHR [20]. In this study, we aim to merge these two lines of studies to validate and extend the positive effect of a patient-framed PFS on users’ decision to upload diseases to the EHR click dummy when disease-specific privacy concerns are systematically varied.

### Aim of This Research and Approach

In this study, we test whether displaying a patient-framed PFS, developed in our previous studies [40,44], shortly before the decision to upload a medical report must be made increases the likelihood that users upload medical reports to the EHR for diseases that vary along 2 dimensions: TC and disease-specific stigma. After describing the methods and results, we discuss the implications, reflect on the study’s limitations, and conclude with a reflection on the objective of this study.

## Methods

### Ethical Considerations

This study was approved by the Ethics Committee of the Department of Psychology and Ergonomics at Technische Universität Berlin (tracking number: AWB_KAL_1_230206_Erweiterungsantrag). The study is registered as a randomized controlled trial at Deutsches Register Klinischer Studien (DRKS00033652). Participants volunteered to participate in the survey, and written informed consent was required to participate. On the first page of the survey, participants were told about the experimenter, the study purpose, what data were to be collected during the study, and where and for how long they would be stored. Also, participants had the possibility to download a PDF with the study information. Hence, participants were informed about the duration of the survey (approximately 8 min) as well as the compensation for participation. All data were collected and stored in an anonymized form. No directly identifying personal information was collected. Data were processed confidentially and used exclusively for research purposes.

### Participants

The online study was conducted between April 15, 2024, and May 16, 2024. Based on an a priori power analysis for a logistic regression using G*Power (version 3.1.9.7) with disease-related stigma (high vs low), TC (acute vs chronic), and privacy information (PFS vs no PFS) as binomial distributed predictors, a false positive rate *α* of .05, a power *β* of .80, an estimated odds ratio (OR) for the predictor with the smallest expected effect size (TC) of 1.7 (derived from the prestudy with n=80 participants), and a probability of the outcome (upload decision) under the null hypothesis of 0.5, reflecting a conservative assumption, we aimed for a sample size of 363 participants. To ensure this target was met, we oversampled participants by 30%, resulting in a total sample of 471 individuals. Oversampling accounted for potential exclusions due to failed attention checks, study dropouts, self-reported invalid data (approximately 20%), as indicated in preliminary studies [40,44], and prior medical histories with the diseases used in the study (approximately 10%, based on prior findings) [18]. Individuals 18 years and older residing in Germany were allowed to participate in the study, as the content and questions of the study were designed to fit the context of the German EHR. Another prerequisite was that participants had no personal previous experience (own illness) with the diseases mentioned in the medical reports we used for this study, as the handling of stigmatized diseases by affected persons is different from that of unaffected persons [45]. Sampling was conducted through Prolific, a crowdsourcing platform used to recruit participants for online surveys and experiments, known for its diverse participant pool and high data quality [46]. Participation was compensated with 1.78€ (US $2.09) for 8 minutes, which corresponds to the German minimum wage. The mean value of the processing time was 8:47 minutes (SD 3:57 min), and the median was 8 minutes. A total of 471 individuals participated in the study.

### Design

The experimental design replicates the disease-related manipulations from our previous study [18] and extends them by testing whether transparent privacy information can reduce disease-related privacy concerns and influence upload behavior. This approach also extends prior privacy calculus–based work by focusing on actual behavior rather than intentions, as behavioral measures provide stronger ecological validity and better capture situational influences on privacy decisions [47,48]. We used a 2×2×2 between-subject study design with the 3 independent variables (IVs): stigmatization potential (SP), TC, and privacy information. Each participant was assigned to one unique combination of these conditions. As in preliminary studies, SP (high vs low) and TC (acute vs chronic) were manipulated by displaying the diagnoses of a disease with the respective characteristics [18,19]. Additionally, privacy information (PFS vs no PFS) was manipulated by either displaying a PFS during the upload process or not. In preliminary studies, participants associate disease-related stigma with high risks [18,19], and consequences could arise in areas related to personal lifestyle, occupation, and social life if medical findings became known [19,45,49,50]. Furthermore, previous studies show that participants perceive the upload of diseases with a chronic TC as more beneficial than the upload of acute diseases [8,19,20]. Participants were randomly (single-blinded) assigned to one of the conditions in parallel (simple randomization, ratio: 1:1:1:1:1:1:1:1) using LimeSurvey’s built-in “rand” function. The randomization process was fully automated within LimeSurvey, ensuring allocation concealment throughout data collection. Neither the participants nor the researchers conducting the data collection were aware of the assigned condition at any point prior to or during data collection. The dependent variable was the decision to upload the medical report, that is, whether participants were willing to upload the medical findings to the EHR [18,19,40]. Reporting of this randomized controlled trial followed the CONSORT-eHEALTH (Consolidated Standards of Reporting Trials of Electronic and Mobile Health Applications and Online Telehealth) guidelines; a completed CONSORT-eHEALTH checklist is provided in Checklist 1.

### Materials

Following a common practice in technology acceptance studies [51,52], we used a case vignette to represent a typical situation in which an EHR app may be used. In particular, the case vignette depicted a situation where the participant has recently started using an EHR app and is now faced with the decision to upload a medical finding to their EHR (see Multimedia Appendix 1). Additionally, the disease/injury was described in lay terms with 1 to 3 sentences (see Multimedia Appendix 2). The stimuli used in the study were realistic but specially created for the purpose of the study. The medical reports were provided by hospitals and a medical association. To make the reports appear as realistic as possible, they were edited on the official document heads of these institutions. This was done with the permission of the institutions concerned. In selecting the diseases, both the related stigma and their TC were systematically varied. Disease-related stigma covered different risks for professional and social life, such as tests for sexually transmitted diseases (ie, gonorrhea and HIV) [53-56] and fractures or rheumatoid arthritis as diseases with low stigma. To reflect different TCs, diseases were divided according to an acute TC (eg, wrist fracture and gonorrhea) and a chronic one (eg, rheumatoid arthritis and HIV). Furthermore, diseases were selected to occur regardless of age, meaning they can affect individuals across different age groups, so that they would be perceived as realistic diseases by an age-diverse sample. Table 1 shows the diseases used as stimuli, categorized by level of perceived stigma and TC.

**Table 1.** Diseases used as stimuli, categorized by SP^a^ and TC^b^.

SP and TC	Acute	Chronic
Low	Fractured wrist	Rheumatoid arthritis
High	Gonorrhea	HIV infection

a SP: stigmatization potential.

b TC: time course.

As in a previous study [19], an interactive prototype (a so-called click dummy) was used, which we created after the mobile EHR app of a German health insurance company (the BARMER) using software for interface design (FIGMA). This prototype allows for a realistic interaction with an EHR. Specifically, the prototype gave participants the ability to upload findings, grant or revoke permissions to view findings, and create medication plans. Only the “Upload findings” function was used in this study.

**Figure 1.**
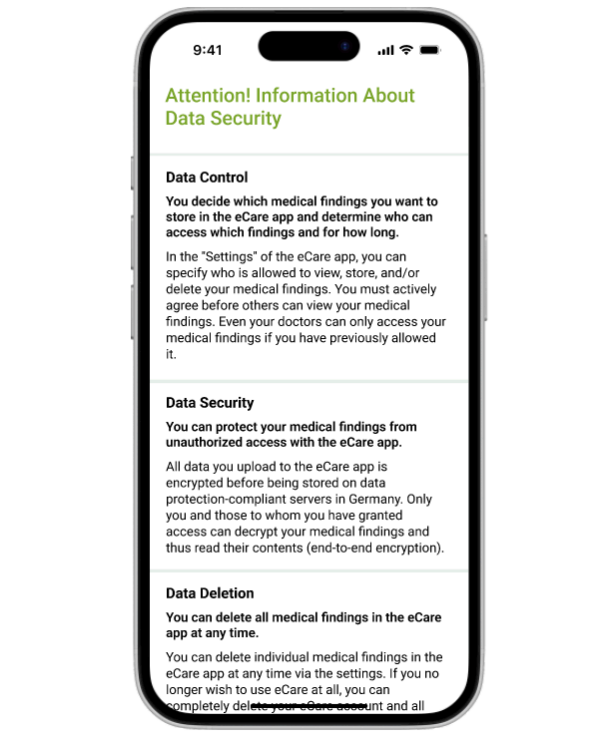
Privacy fact sheets used in the study.

We used the most effective PFS that we identified based on preliminary studies [40,44], which was marked by a concise but comprehensive content and a patient-centered framing, that is, a description of what the EHR allows its users to do to control their data (eg, you can control all of your data) rather than what it does for them (eg, the EHR keeps all of your data safe). Figure 1 shows the English version of the PFS used. The English translation of the full text can be found in the Multimedia Appendix 3.

We used LimeSurvey (version 3.28.66+230719) to create and conduct a 9-page online survey. The EHR prototype was embedded into the survey using iFrame. LimeSurvey software was used to ensure that all questions had to be answered to complete the study and receive the compensation. As in previous studies, we tested the effect of the IVs by querying the perceived risk and perceived benefit of uploading findings to the EHR using validated items [18-20]. Also, we assumed that people perceived more risk when the SP was high and more benefit when the TC was chronic [19,20]. Perceived risk and perceived benefit were measured using a 7-point Likert scale ranging from 1 (“Strongly disagree”) to 7 (“Strongly agree”). The decision to upload the finding was measured using a validated dichotomous item (yes/no) [18,19].

### Procedure

**Figure 2.**
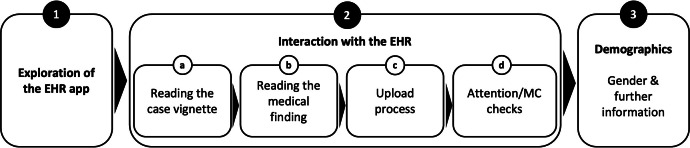
Overview of the study design. EHR: electronic health record; MC: manipulation check.

The study procedure is shown in Figure 2. The survey consisted of 3 parts. After giving their written informed consent according to the Declaration of Helsinki of the World Medical Association, (1) participants had several minutes to interact with the EHR prototype. (2a) Participants then read a randomly selected case vignette addressing the use of the EHR in the context of uploading a medical finding. (2b) Additionally, the participants read the medical findings of the respective illness (low or high SP and acute or chronic TC, depending on the experimental group), as well as a brief description of the respective disease. (2c) Afterwards, as part of the upload process, the participants were asked to select the medical finding for upload. Depending on the experimental group, either a PFS was displayed before the disease could be selected or not. Participants then decided whether they wanted to upload the report to their EHR. (2d) After uploading, participants who were shown a PFS were asked a question about the content of the texts to ensure that the texts were read (attention check), and all participants were asked about the perceived privacy risks and benefits of uploading the report (manipulation check). (3) The survey was completed with the collection of demographic characteristics (age, gender, education level, and experience with mobile health [mHealth] apps) as control variables, as well as the opportunity for participants to declare their responses invalid due to lack of care in processing them (see Multimedia Appendix 4 for the questionnaire).

### Hypotheses

As mentioned above, we hypothesize that diseases with high stigma would result in a high perceived risk and a chronic TC in a high perceived benefit. Hence, we hypothesize that the upload decision is negatively influenced by high disease stigma (H1) and positively influenced by a chronic TC (H2). Based on previous studies, we also assume that a PFS will generally increase upload behavior compared to the no PFS condition (H3). More specifically, regarding the aim of our study, if a PFS can mitigate disease-related concerns, we hypothesize that showing a PFS mitigates the negative influence of disease-related stigma on the upload decision (H4). Furthermore, we hypothesize that the positive influence of a chronic TC on the upload decision will be enhanced by the presence of a PFS, as it enhances perceived benefits related to long-term health management (H5). Textbox 1 provides an overview of the hypotheses regarding the IVs.

Textbox 1. **Overview of the hypotheses.**

The following were the hypotheses regarding the independent variables:

- H1: The number of uploads of medical findings to the electronic health record (EHR) is lower for diseases that are stigmatized compared to those that are nonstigmatized.
- H2: The number of uploads of medical findings to the EHR is higher for chronic diseases compared to acute diseases.
- H3: The number of uploads of medical findings to the EHR is higher when transparency regarding data privacy and security is high compared to when it is low.
- H4: The increase in the number of uploads when showing a privacy fact sheet (PFS) is higher for stigmatized diseases than for nonstigmatized diseases.
- H5: The increase in the number of uploads when showing a PFS is higher for chronic diseases than for acute diseases.

### Analyses

We cleaned and analyzed the data using RStudio (version 2023.09.1+494). The analysis regarding the manipulation checks of perceived privacy risks and benefits was performed using *t* tests, a statistical method used to compare the means of 2 groups. The influence of the IVs (disease-specific stigma, TC, displaying a PFS) and the interaction effects between stigma and the display of a PFS, as well as between TC and the display of a PFS on the upload decision, were tested using multiple logistic regression with dummy coding, a method used to model the probability of a binary outcome based on one or more predictor variables.

We also included a robustness check of the results regarding the upload decision. To control for potential influences of demographic and interindividual variables that could bias coefficients and *P* values, we used multiple logistic regression. To not bias *P* values as a result of controlling, we only included variables in the model that have been shown to have a causal relationship with the IVs (ie, causal confounders): age, education level, and experience with the technical system [38,57,58]. *P* values were adjusted for multiple testing using the Benjamini-Hochberg procedure [59].

## Results

### Survey Characteristics

**Figure 3.**
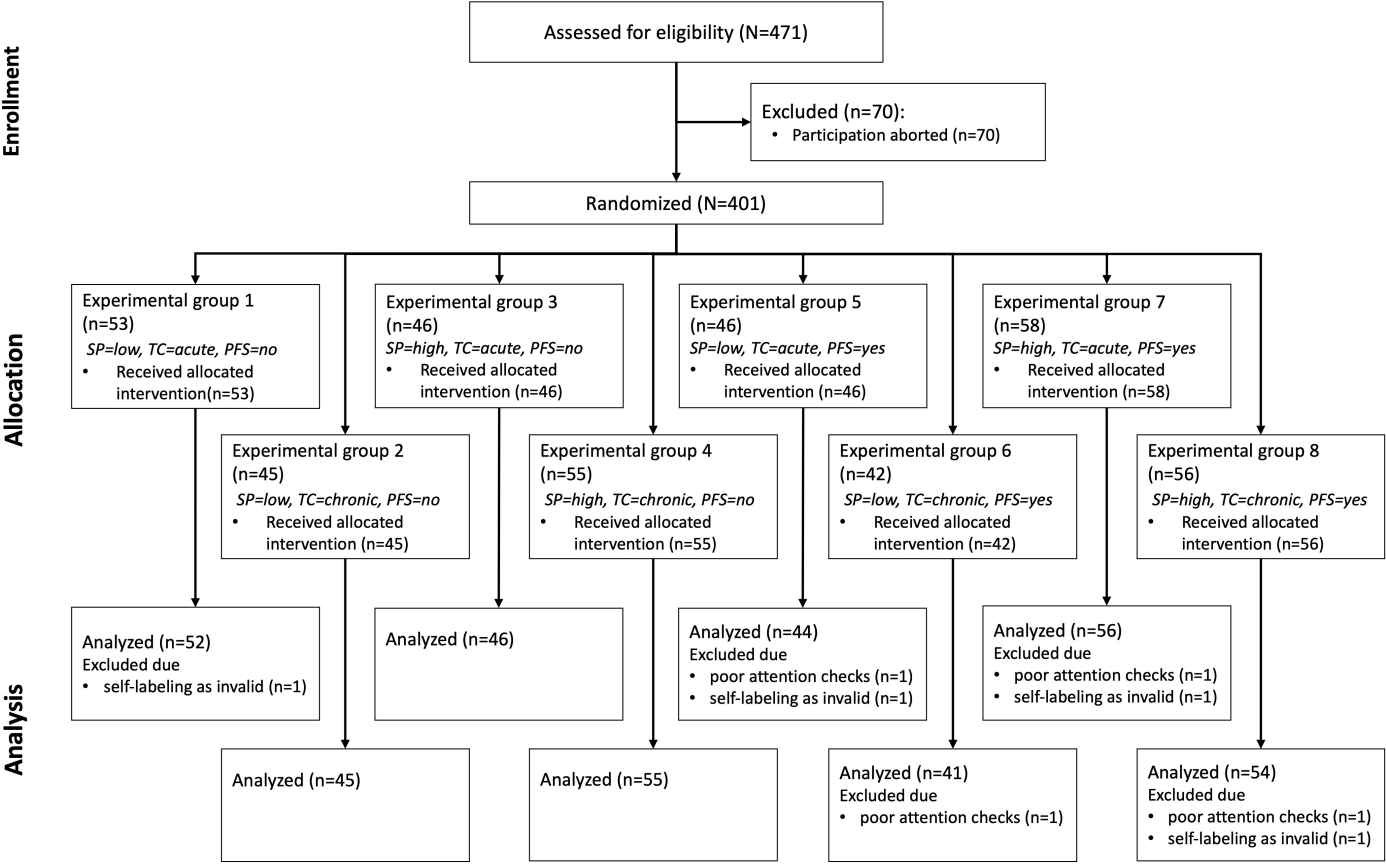
CONSORT (Consolidated Standards of Reporting Trials) flow chart. PFS: privacy fact sheet; SP: stigma potential; TC: time course.

A total of 471 observations were collected. A total of 78 (16.5%) records were excluded, of which 70 (14.9%) were excluded because of incomplete questionnaires, 4 (0.85%) because participants failed the attention check, and 4 (0.85%) because responses were marked as invalid by participants. A sample of 393 observations (156 female participants, 231 male participants, and 6 with no information) was used for further analysis. Figure 3 shows the participation and distribution process according to the guidelines of the CONSORT (Consolidated Standards of Reporting Trials) statement [60].

Table 2 summarizes the demographic characteristics of the entire sample. The demographic characteristics of the subsamples for each experimental group are shown in the Multimedia Appendix 5.

**Table 2.** Demographic data of the sample (n=393).

Demographic characteristic	Respondents
Age (y), mean (SD)	31.67 (9.94)
Sex, n (%)	
Female	156 (39.7)
Male	231 (58.8)
No answer	6 (1.5)
Education, n (%)	
No degree	11 (2.8)
High school/vocational education	179 (45.5)
Bachelor	102 (26.0)
Master	90 (22.9)
PhD	11 (2.8)
Experience with mHealth^a^ apps, n (%)	
No use	226 (57.5)
Regular use	167 (42.5)

a mHealth: mobile health.

### Risk and Benefit Perception

Similar to the preliminary studies [18,19], risk and benefit perception of uploading served as a manipulation check to test the validity of our manipulation (ie, the medical reports) with respect to the perception of risk (stigma) and benefit (TC). As expected, uploading medical findings of stigmatized diseases was perceived as riskier than those of nonstigmatized diseases (low: mean 3.88, SD 1.68; high: mean 5.15, SD 1.6; *t*_391_=7.648; *P*<.001). Consequently, we assume that our risk manipulation was successful. There was no significant difference in the perceived benefit regarding the TC of the disease (acute: mean 5.63, SD 1.41; chronic: mean 5.72, SD 1.16; *t*_391_=0.703; *P*=.483). Consequently, we assume that our benefit manipulation was not successful.

Additionally, we analyzed the benefit perception in relation to stigma and the risk perception in relation to TC, even though these were not part of the initial manipulation checks. Uploading medical findings of nonstigmatized diseases was perceived as more beneficial than those of stigmatized diseases (low: mean 5.84, SD 1.16; high: mean 5.54, SD 1.39; *t*_391_=2.345; *P*=.02). Furthermore, uploading medical findings of chronic diseases into the EHR was perceived as riskier than those of acute diseases (acute: mean 4.31, SD 1.83; chronic: mean 4.82, SD 1.64; *t*_391_=2.893; *P*=.004).

### Upload Behavior

**Figure 4.**
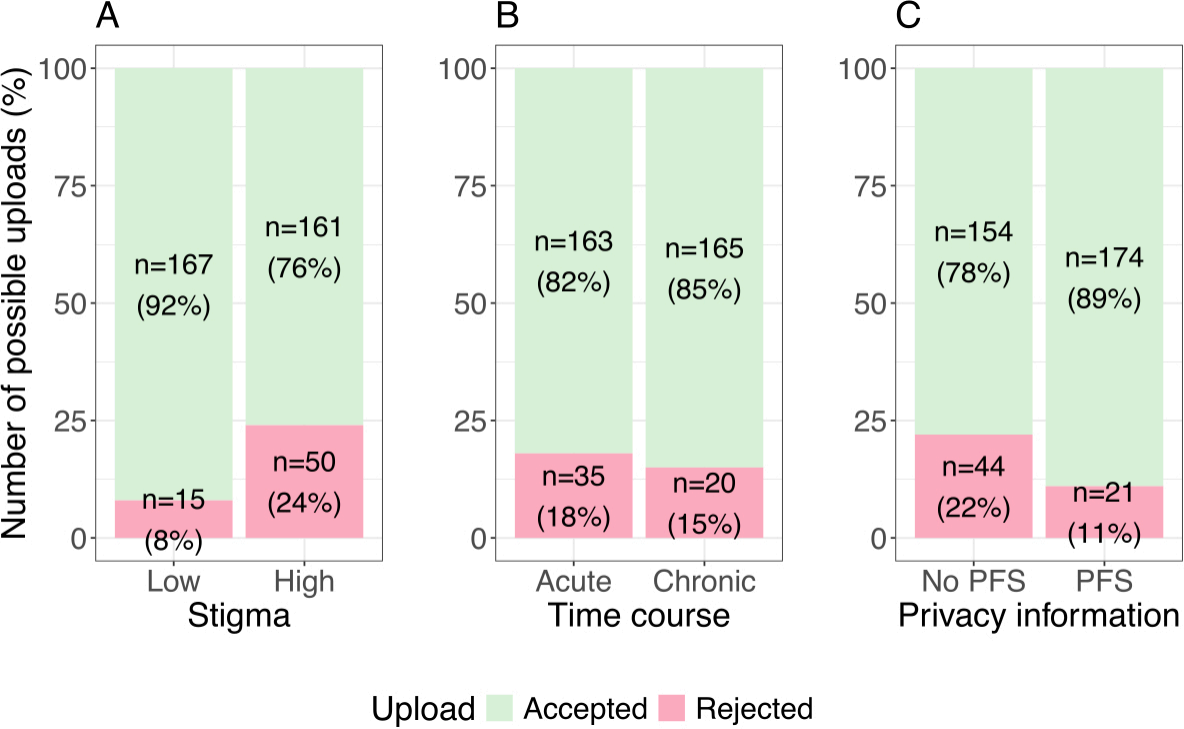
Number of uploads to the electronic health record as a function of disease-related (A) stigma, (B) time course, and (C) privacy information. PFS: privacy fact sheet.

Upload behavior was negatively associated with disease-related stigma (*z*=4.568; *P*<.001), thus supporting H1. Specifically, when stigma was high, it was more than seven times less likely that the report was uploaded to the EHR (76.3%, 161/211) than when stigma was low (91.8%, 167/182). TC of the disease was not associated with the decision to upload a report (z=0.877; *P*=.38). Consequently, H2 is rejected. The PFS was positively associated with the decision to upload a medical report to the EHR (*z*=3.298; *P*<.001), supporting H3. When a PFS was given, participants were more than 4 times as likely to upload the diagnosis to their EHR (89.2%, 174/195) than when a PFS was not given (77.7%, 154/198). The absolute number of uploads is shown in Figure 4 as a function of the IVs disease-related stigma, TC, and PFS vs no PFS.

**Figure 5.**
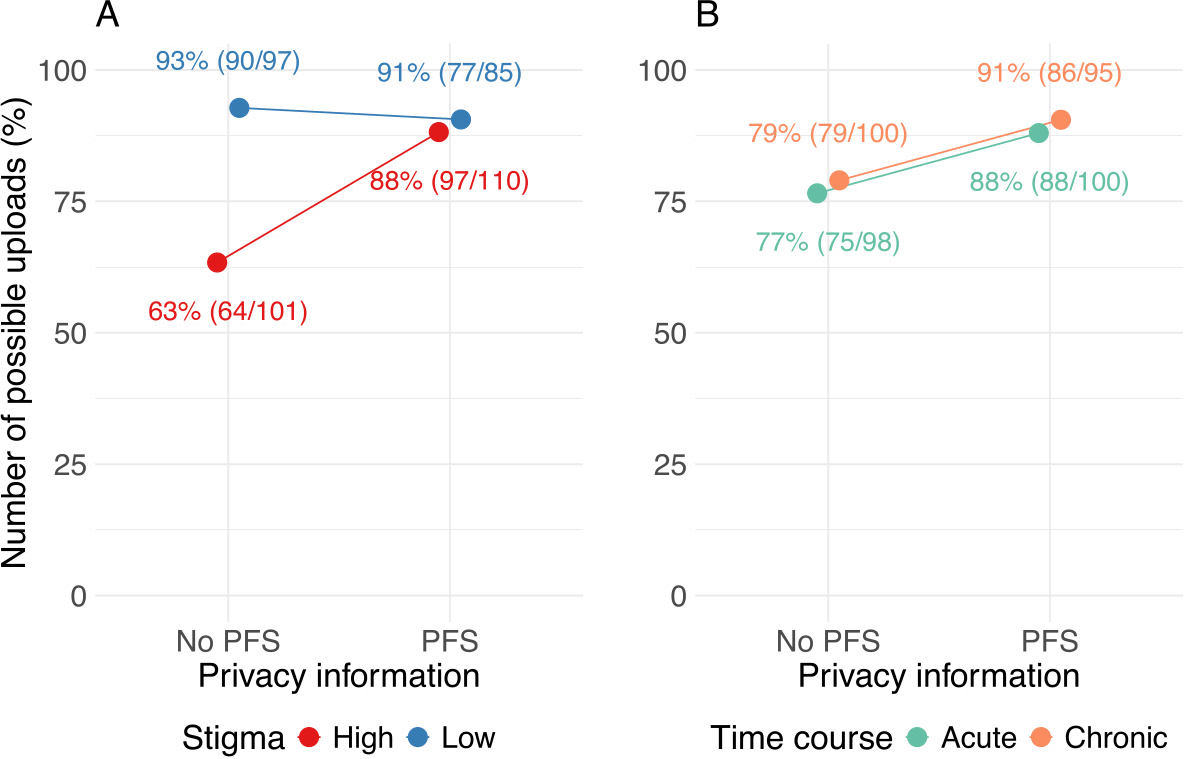
Number of uploads to the electronic health record as interaction between stigma and privacy information (A) and time course and privacy information (B). PFS: privacy fact sheet.

We also tested for interaction effects between stigma and privacy information, as well as between TC and privacy information, to explore potential moderating effects. The interaction between stigma and privacy information was significant (*z*=2.734; *P*=.006), indicating that the increase in the number of uploads when showing a PFS is higher for stigmatized diseases than for nonstigmatized diseases, thus supporting H4 (see Figure 5A). In contrast, the interaction between TC and privacy information was not significant (*z*=0.094; *P*=.92), suggesting that displaying a PFS did not differentially impact the upload decision based on whether the disease was acute or chronic (see Figure 5B). Consequently, H5 is rejected. The summary of the results of the logistic regression is shown in Table 3.

**Table 3.** Results of the logistic regression.^a^

Variable	z value	*P* value	ORs^b^ and 95% CIs		
			Lower	OR	Upper
Stigma (high vs low)	4.568	<.001	0.050	0.130	0.296
TC^c^ (acute vs chronic)	0.877	.38	0.672	1.382	2.869
Privacy information (PFS^d^ vs no PFS)	3.298	<.001	1.888	4.527	11.490
Stigma*privacy information	2.734	.006	1.661	5.952	21.739
TC*privacy information	0.094	.92	0.325	1.058	3.390

a *R*2=0.107 (Hosmer-Lemeshow), 0.297 (Nagelkerke), 0.284 (Cox-Snell). Model *χ*2_5_=37.68; *P*<.001.

b OR: odds ratio.

c TC: time course.

d PFS: privacy fact sheet.

### Robustness Check

When controlling for interindividual variables (age, gender, education, and mHealth experience), the effects of stigma (*z*=4.820; *P*<.001) and information transparency (*z*=3.548; *P*<.001) and their interaction (*z*=3.086; *P*=.002) remained robust. Age had a negative effect on the upload behavior (*z*=2.531; OR 0.965, 95% CI 0.939‐0.992; *P*=.01). With an increase in age, users were less likely to upload medical findings into their EHR. The other control variables did not influence the upload behavior.

## Discussion

### Principal Findings

**Figure 6.**
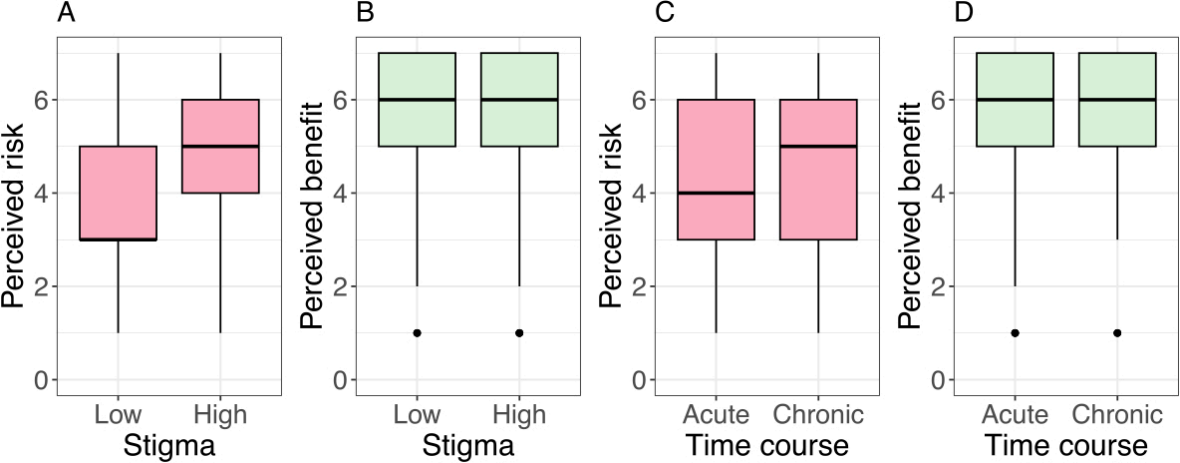
Perceived risk as a function of disease-related (A) stigma and (C) time course, and perceived benefit as a function of (B) stigma and (D) time course. The horizontal line in the box represents the median.

The results of our study show that the decision to upload an individual medical report to an EHR click dummy is influenced by disease-related stigma as well as by privacy notices, that is, concise but comprehensive information about data privacy choices and security measures in the form of PFS. As in our preliminary studies [18,19], uploading diseases with high stigma was associated with increased privacy risk perceptions compared to diseases with low stigma (see Figure 6A), which increased the likelihood of rejecting uploads for stigmatized diseases 6 fold compared to nonstigmatized diseases (see Figure 4A). This finding is surprising given a generally high rating of potential benefits of uploading reports to the EHR (see Figure 6B D) and an overall high willingness to upload medical findings to the EHR. In addition to the nonsignificant manipulation check regarding perceived benefits, uploads did not vary with the TC of the disease (see Figure 4).

Furthermore, a PFS positively influenced the decision to upload. When a PFS was displayed, the likelihood of uploading medical findings to the EHR was more than 3 times higher than when it was not given (see Figure 4C). This is in line with the findings of various studies showing that effective communication of data privacy choices and security information enables people to make informed decisions, thereby reducing general privacy concerns and increasing the use of EHRs [7,24,33,35,61,62]. A plausible explanation for this effect is that a PFS increases users’ perceived control over personal data—allowing them to understand and oversee how their health data are handled—which in turn reduces perceived risks and facilitates the decision to upload their data [7,38,40].

Displaying a PFS did not influence the decision to upload medical findings for nonstigmatized diseases, as nearly all nonstigmatized medical reports were uploaded regardless of whether a PFS was given (see Figure 5A). In contrast, for stigmatized diseases, the PFS significantly increased the likelihood of uploads (see Figure 5B). This suggests that showing a PFS shortly before a decision to upload medical findings to the EHR must be made not only is effective in mitigating general privacy concerns but also helps to reduce specific fears related to stigmatized diseases and increase upload decisions [36,37,40].

More generally, studies in nonmedical domains, involving low-risk scenarios such as a shopping assistant [43] and an event finder [36], showed that transparency features or the transparency of privacy policies had no effect on behavior, for instance, on the decision to access the location [43] or the intention to disclose personal data to the event finder [36]. Our findings help to explain these differing findings by showing that the relevance of transparent privacy notes is mainly contingent upon the level of perceived risk associated with the data. In low-risk scenarios, such as nonstigmatized diseases, privacy concerns are typically low, which means that transparency features cannot meaningfully change the decision because there are no substantial concerns to alleviate. However, in high-risk scenarios, such as those involving stigmatized health conditions, privacy concerns are more likely to arise. In these situations, transparency features can help strengthen users’ perceived control, reducing perceived risks and thereby increasing acceptance. This highlights the importance of situational context for transparency measures to matter. Transparent information about data privacy and security is not necessary in low-risk situations (although it does not hurt behavioral outcomes), but it becomes crucial for decision making in high-risk contexts, such as the handling of sensitive health data in EHRs.

### Implications

The opportunities offered by implementing transparency features in the EHR should be considered by health care stakeholders. Transparency features can not only reduce general privacy concerns but can also address situational concerns triggered by disease-related stigma [18]. Thus, transparency features can ultimately help to ensure equal access to EHRs, even for users who suffer from stigmatized diseases, thereby promoting health equity [49,50,63]. This way, more patients get a chance to benefit from the EHR and, as their illnesses, allergies, and medications can be considered for future diagnostics and therapies, receive better and more targeted treatment.

### Limitations and Future Directions

There are several limitations in our study, which need to be considered in subsequent studies. While our manipulation checks for perceived risk (related to stigma) were successful, the manipulation of perceived benefit (related to TC) was not. This may be due to the between-subjects design of our study. In a previous within-subjects design, where participants evaluated both acute and chronic reports, the TC significantly impacted upload behavior [19]. It seems that participants, when comparing multiple conditions, can better discern when uploading is more or less beneficial. In our study, however, participants may have perceived the benefits of uploading as uniformly high, regardless of TC, leading to a diminished ability to detect differences.

It is clear that the adoption and approval of data-gathering technologies are strongly influenced by cultural differences [64]. In comparison to other European nations, the German population exhibits a heightened level of caution regarding the use of personal information online [65]. Given that in this study, data collection was conducted solely with residents of Germany, future studies should validate the applicability of these findings in other countries.

We deliberately excluded participants who already had a medical history with the diseases addressed in the stimuli to avoid bias in their responses. Individuals living with a stigmatized disease are more cautious to disclose the information, especially if the disease is not immediately apparent [45,66]. The question arises to what extent the behavior of stigmatized individuals can be simulated under experimental conditions. To further strengthen the validity and generalizability of our results, a follow-up study should examine the perspective of already affected individuals.

Although we captured actual click behavior, the upload decision occurred in a simulated EHR environment using fictitious diagnoses and did not involve participants’ actual health data. As such, the behavior measured in this study reflects a behavioral proxy rather than real-world EHR upload behavior. Future work should examine the robustness of these findings and investigate how patients behave when real data, real accounts, and real consequences are involved.

Moreover, this was a survey study with limited immersion despite the use of an interactive click dummy. In a follow-up study, researchers could collaborate with health insurers to gather real-world data on upload behavior with a real EHR and an integrated transparency feature as used for this study. Conversely, our study faced limitations due to uncontrolled conditions like participants’ locations and potential distractions, as participants completed the questionnaire online. Future research could validate our findings through a laboratory study, ensuring a more controlled environment.

Another limitation is that the distribution of our sample in terms of gender, age, and level of education does not correspond to that of the average German population [67,68]. In particular, the level of education of our sample was above average. Although we were unable to detect any effects of the control variables, gender and level of education, in the analysis, the results of this study should be validated with a more representative sample in the future.

### Conclusions

Our results show that although general upload rates to the EHR are high, stigmatized diseases—even if simulated—negatively affect simulated upload behavior. However, displaying a transparency feature in the form of a PFS increases the likelihood that people upload stigmatized health data when interacting with an EHR click dummy by mitigating privacy concerns. Our findings indicate that the role of transparency features is contingent upon the level of perceived risk associated with the data to be uploaded. When the perceived risk is low, users do not need detailed privacy information to trust the technology and upload their data. However, when uploads involve sensitive data and are seen as risky, users consider privacy information and modify their upload behavior based on the information provided, potentially because transparency features help strengthen users’ perceived control in such high-risk situations. Implementing transparency features in EHRs may thus help to ensure that users who perceive high privacy risks when confronted with sensitive health information are not excluded from the benefits of these systems due to privacy concerns, thereby promoting digital health equity.

## Supplementary material

10.2196/71124 Multimedia Appendix 1 Case vignettes.

10.2196/71124 Multimedia Appendix 2 Disease descriptions.

10.2196/71124 Multimedia Appendix 3 Privacy fact sheets.

10.2196/71124 Multimedia Appendix 4 Questionnaire.

10.2196/71124 Multimedia Appendix 5 Demographic data of all groups.

10.2196/71124 Checklist 1 CONSORT-EHEALTH (v 1.6.1) checklist.

## Abbreviations

CONSORT: Consolidated Standards of Reporting Trials
CONSORT-EHEALTH: Consolidated Standards of Reporting Trials of Electronic and Mobile Health Applications and Online Telehealth
EHR: electronic health record
IV: independent variable
mHealth: mobile health
OR: odds ratio
PFS: privacy fact sheet
SP: stigmatization potential
TC: time course
