# Supplementary material for: Privacy Fact Sheets for Mitigating Disease-Related Privacy Concerns and Facilitating Equal Access to the Electronic Health Record: Randomized Controlled Trial
Source: JMIR Hum Factors. 2026 Jan 15;13:e71124. doi: 10.2196/71124 (PMC12806596; doi:10.2196/71124)
Supplement: Multimedia Appendix 3 [file humanfactors-v13-e71124-s003.pdf]

Privacy fact sheet

Attention! Information About Data Security

Data Control

You decide which medical findings you want to store in the eCare app and determine who can access which findings and for how long.

In the "Settings" of the eCare app, you can specify who is allowed to view, store, and/or delete your medical findings. You must actively agree before others can view your medical findings. Even your doctors can only access your medical findings if you have previously allowed it.

Data Security

You can protect your medical findings from unauthorized access with the eCare app.

All data you upload to the eCare app is encrypted before being stored on data protection-compliant servers in Germany. Only you and those to whom you have granted access can decrypt your medical findings and thus read their contents (end-to-end encryption).

Data Deletion

You can delete all medical findings in the eCare app at any time.

You can delete individual medical findings in the eCare app at any time via the settings. If you no longer wish to use eCare at all, you can completely delete your eCare account and all your medical findings. Simply revoke your consent to the privacy and terms of use by email to service@barmer.de.
