# Supplementary material for: Privacy Fact Sheets for Mitigating Disease-Related Privacy Concerns and Facilitating Equal Access to the Electronic Health Record: Randomized Controlled Trial
Source: JMIR Hum Factors. 2026 Jan 15;13:e71124. doi: 10.2196/71124 (PMC12806596; doi:10.2196/71124)
Supplement: Multimedia Appendix 5 [file humanfactors-v13-e71124-s005.pdf]

Table 1. Demographic data of the sample regarding all experimental groups (N=393). Note: SP = stigma potential, TC = time course, PFS= privacy fact sheet

**Demographic characteristic**	**Low SP, acute TC, no PFS (n=52)**	**Low SP, chronic TC, no PFS (n=45)**	**High SP, acute TC, no PFS (n=46)**	**High SP, chronic TC, no PFS (n=55)**	**Low SP, acute TC, with PFS (n=44)**	**Low SP, chronic TC, with PFS (n=41)**	**High SP, acute TC, with PFS (n=56)**	**High SP, chronic TC, with PFS (n=54)**	**Total (N=393)**
Age (years), mean (SD)	30.04 (7.18)	32.18 (11.14)	31.11 (11.09)	33.04 (10.68)	30.50 (9.62)	31.07 (9.09)	31.54 (9.41)	32.72 (10.11)	31.67 (9.94)
**Gender, n (%)**									
female	20 (38.5)	22 (48.9)	18 (39.1)	26 (47.3)	15 (34.1)	15 (36.6)	19 (33.9)	21 (38.9)	156 (39.7)
male	30 (57.7)	22 (48.9)	27 (58.7)	29 (52.7)	28 (63.6)	25 (61.0)	37 (66.1)	33 (61.1)	231 (58.8)
no answer	2 (3.8)	1 (2.2)	1 (2.2)	0 (0)	1 (2.3)	1 (2.4)	0 (0)	0 (0)	6 (1.5)
**Education, n (%)**									
No degree	0 (0)	2 (4.4)	0 (0)	4 (7.3)	1 (2.3)	2 (4.9)	1 (1.8)	1 (1.9)	11 (2.8)
Highschool / vocational education	29 (55.8)	17 (37.8)	20 (43.5)	27 (49.1)	20 (45.5)	19 (46.3)	24 (42.9)	23 (42.6)	179 (45.5)
Bachelor	12 (23.1)	12 (26.7)	12 (26.1)	15 (27.3)	12 (27.3)	12 (29.3)	15 (26.8)	12 (22.2)	102 (26.0)
Master	10 (19.2)	8 (17.8)	14 (30.4)	9 (16.4)	9 (20.5)	8 (19.5)	14 (25.0)	18 (33.3)	90 (22.9)
PhD	1 (1.9)	6 (13.3)	0 (0)	0 (0)	2 (4.5)	0 (0)	2 (3.6)	0 (0)	11 (2.8)
**Experience with mHealth applications, n (%)**									
No use	26 (50.0)	29 (64.4)	27 (58.7)	34 (61.8)	24 (54.5)	24 (58.5)	28 (50.0)	36 (66.7)	226 (57.5)
Regular use	26 (50.0)	16 (35.6)	27 (41.3)	21 (38.2)	20 (45.5)	17 (41.5)	28 (50.0)	18 (33.3)	167 (42.5)
