# Supplementary material for: Privacy Fact Sheets for Mitigating Disease-Related Privacy Concerns and Facilitating Equal Access to the Electronic Health Record: Randomized Controlled Trial
Source: JMIR Hum Factors. 2026 Jan 15;13:e71124. doi: 10.2196/71124 (PMC12806596; doi:10.2196/71124)
Supplement: Checklist 1 [file humanfactors-v13-e71124-s006.pdf]

CONSORT-EHEALTH (V 1.6.1) - Submission/Publication Form

The CONSORT-EHEALTH checklist is intended for authors of randomized trials evaluating web-based and Internet-based applications/interventions, including mobile interventions, electronic games (incl multiplayer games), social media, certain telehealth applications, and other interactive and/or networked electronic applications. Some of the items (e.g. all subitems under item 5 - description of the intervention) may also be applicable for other study designs.

The goal of the CONSORT EHEALTH checklist and guideline is to be
a) a guide for reporting for authors of RCTs,
b) to form a basis for appraisal of an ehealth trial (in terms of validity)

CONSORT-EHEALTH items/subitems are MANDATORY reporting items for studies published in the Journal of Medical Internet Research and other journals / scientific societies endorsing the checklist.

Items numbered 1., 2., 3., 4a., 4b etc are original CONSORT or CONSORT-NPT (non-pharmacologic treatment) items.
Items with Roman numerals (i., ii, iii, iv etc.) are CONSORT-EHEALTH extensions/clarifications.

As the CONSORT-EHEALTH checklist is still considered in a formative stage, we would ask that you also RATE ON A SCALE OF 1-5 how important/useful you feel each item is FOR THE PURPOSE OF THE CHECKLIST and reporting guideline (optional).

Mandatory reporting items are marked with a red *.
In the textboxes, either copy & paste the relevant sections from your manuscript into this form - please include any quotes from your manuscript in QUOTATION MARKS,
or answer directly by providing additional information not in the manuscript, or elaborating on why the item was not relevant for this study.

YOUR ANSWERS WILL BE PUBLISHED AS A SUPPLEMENTARY FILE TO YOUR PUBLICATION IN JMIR AND ARE CONSIDERED PART OF YOUR PUBLICATION (IF ACCEPTED).
Please fill in these questions diligently. Information will not be copyedited, so please use proper spelling and grammar, use correct capitalization, and avoid abbreviations.

DO NOT FORGET TO SAVE AS PDF _AND_ CLICK THE SUBMIT BUTTON SO YOUR ANSWERS ARE IN OUR DATABASE !!!

Sie bearbeiten gerade Ihre Antwort. Wenn Sie die URL freigeben, können andere Personen Ihre Antwort ebenfalls bearbeiten.

NEUE ANTWORT AUSFÜLLEN

CONSORT-EHEALTH: Improving and Standardizing Evaluation Reports of Web-based and

Mobile Health Interventions
J Med Internet Res 2011;13(4):e126
URL: http://www.jmir.org/2011/4/e126/
doi: 10.2196/jmir.1923
PMID: 22209829

niklasvk@googlemail.com Konto wechseln

Nicht freigegeben

Formular zum Speichern noch einmal einreiche

* Gibt eine erforderliche Frage an

Your name *

First Last

Niklas von Kalckreuth

Primary Affiliation (short), City, Country *

University of Toronto, Toronto, Canada

Technische Universität Berlin, Berlin, Germany

Your e-mail address *

abc@gmail.com

niklas.vkalckreuth@tu-berlin.de

Title of your manuscript *

Provide the (draft) title of your manuscript.

Privacy Fact Sheets mitigate Disease-related Privacy Concerns and Facilitate Equal Access
[illegible]

Sie bearbeiten gerade Ihre Antwort. Wenn Sie die URL freigeben, können andere Personen Ihre Antwort ebenfalls bearbeiten.

NEUE ANTWORT AUSFÜLLEN

Name of your App/Software/Intervention *

If there is a short and a long/alternate name, write the short name first and add the long name in brackets.

Privacy Fact Sheet (Transparency Feature)

Evaluated Version (if any)

e.g. "V1", "Release 2017-03-01", "Version 2.0.27913"

not applicable

Language(s) *

What language is the intervention/app in? If multiple languages are available, separate by comma (e.g. "English, French")

English

URL of your Intervention Website or App

e.g. a direct link to the mobile app on app in appstore (itunes, Google Play), or URL of the website. If the intervention is a DVD or hardware, you can also link to an Amazon page.

Meine Antwort

URL of an image/screenshot (optional)

Meine Antwort

Sie bearbeiten gerade Ihre Antwort. Wenn Sie die URL freigeben, können andere Personen Ihre Antwort ebenfalls bearbeiten.

NEUE ANTWORT AUSFÜLLEN

Accessibility *

Can an enduser access the intervention presently?

- ( ) access is free and open
- ( ) access only for special usergroups, not open
- ( ) access is open to everyone, but requires payment/subscription/in-app purchases
- (x) app/intervention no longer accessible
- ( ) Sonstiges:

Primary Medical Indication/Disease/Condition *

e.g. "Stress", "Diabetes", or define the target group in brackets after the condition, e.g. "Autism (Parents of children with)", "Alzheimers (Informal Caregivers of)"

Healthy patients

Primary Outcomes measured in trial *

comma-separated list of primary outcomes reported in the trial

Upload behavior, intention to use

Secondary/other outcomes

Are there any other outcomes the intervention is expected to affect?

perceived benefits, perceived risks

Sie bearbeiten gerade Ihre Antwort. Wenn Sie die URL freigeben, können andere Personen Ihre Antwort ebenfalls bearbeiten.

NEUE ANTWORT AUSFÜLLEN

Recommended "Dose" *

What do the instructions for users say on how often the app should be used?

- ○ Approximately Daily
- ○ Approximately Weekly
- ○ Approximately Monthly
- ○ Approximately Yearly
- ◉ "as needed"
- ○ Sonstiges:

Approx. Percentage of Users (starters) still using the app as recommended after 3 months *

- ◉ unknown / not evaluated
- ○ 0-10%
- ○ 11-20%
- ○ 21-30%
- ○ 31-40%
- ○ 41-50%
- ○ 51-60%
- ○ 61-70%
- ○ 71%-80%
- ○ 81-90%
- ○ 91-100%
- ○ Sonstiges:

Sie bearbeiten gerade Ihre Antwort. Wenn Sie die URL freigeben, können andere Personen Ihre Antwort ebenfalls bearbeiten.

NEUE ANTWORT AUSFÜLLEN

Overall, was the app/intervention effective? *

- (x) yes: all primary outcomes were significantly better in intervention group vs control
- ( ) partly: SOME primary outcomes were significantly better in intervention group vs control
- ( ) no statistically significant difference between control and intervention
- ( ) potentially harmful: control was significantly better than intervention in one or more outcomes
- ( ) inconclusive: more research is needed
- ( ) Sonstiges:

Article Preparation Status/Stage *

At which stage in your article preparation are you currently (at the time you fill in this form)

- ( ) not submitted yet - in early draft status
- (x) not submitted yet - in late draft status, just before submission
- ( ) submitted to a journal but not reviewed yet
- ( ) submitted to a journal and after receiving initial reviewer comments
- ( ) submitted to a journal and accepted, but not published yet
- ( ) published
- ( ) Sonstiges:

Sie bearbeiten gerade Ihre Antwort. Wenn Sie die URL freigeben, können andere Personen Ihre Antwort ebenfalls bearbeiten.

NEUE ANTWORT AUSFÜLLEN

Journal *

If you already know where you will submit this paper (or if it is already submitted), please provide the journal name (if it is not JMIR, provide the journal name under "other")

- ○ not submitted yet / unclear where I will submit this
- ○ Journal of Medical Internet Research (JMIR)
- ○ JMIR mHealth and UHealth
- ○ JMIR Serious Games
- ○ JMIR Mental Health
- ○ JMIR Public Health
- ○ JMIR Formative Research
- ◉ Other JMIR sister journal
- ○ Sonstiges:

Is this a full powered effectiveness trial or a pilot/feasibility trial? *

- ○ Pilot/feasibility
- ◉ Fully powered

Manuscript tracking number *

If this is a JMIR submission, please provide the manuscript tracking number under "other" (The ms tracking number can be found in the submission acknowledgement email, or when you login as author in JMIR. If the paper is already published in JMIR, then the ms tracking number is the four-digit number at the end of the DOI, to be found at the bottom of each published article in JMIR)

- ◉ no ms number (yet) / not (yet) submitted to / published in JMIR
- ○ [illegible]

Sie bearbeiten gerade Ihre Antwort. Wenn Sie die URL freigeben, können andere Personen Ihre Antwort ebenfalls bearbeiten.

NEUE ANTWORT AUSFÜLLEN

TITLE AND ABSTRACT

1a) TITLE: Identification as a randomized trial in the title

1a) Does your paper address CONSORT item 1a? *

I.e does the title contain the phrase "Randomized Controlled Trial"? (if not, explain the reason under "other")

- (x) yes
- ( ) Sonstiges:

1a-i) Identify the mode of delivery in the title

Identify the mode of delivery. Preferably use “web-based” and/or “mobile” and/or “electronic game” in the title. Avoid ambiguous terms like “online”, “virtual”, “interactive”. Use “Internet-based” only if Intervention includes non-web-based Internet components (e.g. email), use “computer-based” or “electronic” only if offline products are used. Use “virtual” only in the context of “virtual reality” (3-D worlds). Use “online” only in the context of “online support groups”. Complement or substitute product names with broader terms for the class of products (such as “mobile” or “smart phone” instead of “iphone”), especially if the application runs on different platforms.

	1	2	3	4	5	
subitem not at all important	(x)	( )	( )	( )	( )	essential

Auswahl löschen

Sie bearbeiten gerade Ihre Antwort. Wenn Sie die URL freigeben, können andere Personen Ihre Antwort ebenfalls bearbeiten.

NEUE ANTWORT AUSFÜLLEN

Does your paper address subitem 1a-i? *

Copy and paste relevant sections from manuscript title (include quotes in quotation marks "like this" to indicate direct quotes from your manuscript), or elaborate on this item by providing additional information not in the ms, or briefly explain why the item is not applicable/relevant for your study

No, we used the term "randomized controlled trial"

1a-ii) Non-web-based components or important co-interventions in title

Mention non-web-based components or important co-interventions in title, if any (e.g., “with telephone support”).

	1	2	3	4	5	
subitem not at all important	◉	○	○	○	○	essential

Auswahl löschen

Does your paper address subitem 1a-ii?

Copy and paste relevant sections from manuscript title (include quotes in quotation marks "like this" to indicate direct quotes from your manuscript), or elaborate on this item by providing additional information not in the ms, or briefly explain why the item is not applicable/relevant for your study

not applicable, because the experiment was conducted solely web-based

Sie bearbeiten gerade Ihre Antwort. Wenn Sie die URL freigeben, können andere Personen Ihre Antwort ebenfalls bearbeiten.

NEUE ANTWORT AUSFÜLLEN

1a-iii) Primary condition or target group in the title

Mention primary condition or target group in the title, if any (e.g., “for children with Type I Diabetes”) Example: A Web-based and Mobile Intervention with Telephone Support for Children with Type I Diabetes: Randomized Controlled Trial

	1	2	3	4	5	
subitem not at all important	(●)	○	○	○	○	essential

Auswahl löschen

Does your paper address subitem 1a-iii? *

Copy and paste relevant sections from manuscript title (include quotes in quotation marks "like this" to indicate direct quotes from your manuscript), or elaborate on this item by providing additional information not in the ms, or briefly explain why the item is not applicable/relevant for your study

none applicable, because this study did not targeted special groups

1b) ABSTRACT: Structured summary of trial design, methods, results, and conclusions

NPT extension: Description of experimental treatment, comparator, care providers, centers, and blinding status.

Sie bearbeiten gerade Ihre Antwort. Wenn Sie die URL freigeben, können andere Personen Ihre Antwort ebenfalls bearbeiten.

NEUE ANTWORT AUSFÜLLEN

1b-i) Key features/functionalities/components of the intervention and comparator in the METHODS section of the ABSTRACT

Mention key features/functionalities/components of the intervention and comparator in the abstract. If possible, also mention theories and principles used for designing the site. Keep in mind the needs of systematic reviewers and indexers by including important synonyms. (Note: Only report in the abstract what the main paper is reporting. If this information is missing from the main body of text, consider adding it)

	1	2	3	4	5	
subitem not at all important	○	○	○	○	◉	essential

Auswahl löschen

Does your paper address subitem 1b-i? *

Copy and paste relevant sections from the manuscript abstract (include quotes in quotation marks "like this" to indicate direct quotes from your manuscript), or elaborate on this item by providing additional information not in the ms, or briefly explain why the item is not applicable/relevant for your study

In an online user study, 393 German participants were asked to interact with a randomly assigned medical report that varied systematically in terms of disease-related stigma (high vs. low) and time course (acute vs. chronic). They were then asked to decide whether to upload the report to the EHR, while we systematically varied the presentation of privacy information (PFS vs. no PFS).

1b-ii) Level of human involvement in the METHODS section of the ABSTRACT

Clarify the level of human involvement in the abstract, e.g., use phrases like “fully automated” vs. “therapist/nurse/care provider/physician-assisted” (mention number and expertise of providers involved, if any). (Note: Only report in the abstract what the main paper is reporting. If this information is missing from the main body of text, consider adding it)

	1	2	3	4	5	
subitem not at all [illegible]	◉	○	○	○	○	essential

Sie bearbeiten gerade Ihre Antwort. Wenn Sie die URL freigeben, können andere Personen Ihre Antwort ebenfalls bearbeiten.

NEUE ANTWORT AUSFÜLLEN

Does your paper address subitem 1b-ii?

Copy and paste relevant sections from the manuscript abstract (include quotes in quotation marks "like this" to indicate direct quotes from your manuscript), or elaborate on this item by providing additional information not in the ms, or briefly explain why the item is not applicable/relevant for your study

Not applicable, because we did not directly evaluate a system. Participants just had to decide to upload medical findings to the EHR

1b-iii) Open vs. closed, web-based (self-assessment) vs. face-to-face assessments in the METHODS section of the ABSTRACT

Mention how participants were recruited (online vs. offline), e.g., from an open access website or from a clinic or a closed online user group (closed usergroup trial), and clarify if this was a purely web-based trial, or there were face-to-face components (as part of the intervention or for assessment). Clearly say if outcomes were self-assessed through questionnaires (as common in web-based trials). Note: In traditional offline trials, an open trial (open-label trial) is a type of clinical trial in which both the researchers and participants know which treatment is being administered. To avoid confusion, use “blinded” or “unblinded” to indicated the level of blinding instead of “open”, as “open” in web-based trials usually refers to “open access” (i.e. participants can self-enrol). (Note: Only report in the abstract what the main paper is reporting. If this information is missing from the main body of text, consider adding it)

	1	2	3	4	5	
subitem not at all important	○	○	○	○	◉	essential

Auswahl löschen

Does your paper address subitem 1b-iii?

Copy and paste relevant sections from the manuscript abstract (include quotes in quotation marks "like this" to indicate direct quotes from your manuscript), or elaborate on this item by providing additional information not in the ms, or briefly explain why the item is not applicable/relevant for your study

In an online user study, 393 German participants from the recruitment platform Prolific were

Sie bearbeiten gerade Ihre Antwort. Wenn Sie die URL freigeben, können andere Personen Ihre Antwort ebenfalls bearbeiten.

NEUE ANTWORT AUSFÜLLEN

1b-iv) RESULTS section in abstract must contain use data

Report number of participants enrolled/assessed in each group, the use/uptake of the intervention (e.g., attrition/adherence metrics, use over time, number of logins etc.), in addition to primary/secondary outcomes. (Note: Only report in the abstract what the main paper is reporting. If this information is missing from the main body of text, consider adding it)

	1	2	3	4	5	
subitem not at all important	○	○	○	○	◉	essential

Auswahl löschen

Does your paper address subitem 1b-iv?

Copy and paste relevant sections from the manuscript abstract (include quotes in quotation marks "like this" to indicate direct quotes from your manuscript), or elaborate on this item by providing additional information not in the ms, or briefly explain why the item is not applicable/relevant for your study

Participants were randomly (single-blinded) assigned to one of the 8 conditions in parallel (stigma, time course, privacy information) : low, acute, no PFS (52/393, 13.2%), low, chronic, no PFS (45/393, 11.5%), high, acute, no PFS (46/393, 11.7%), high, chronic, no PFS (55/393, 14%), low, acute, PFS (44/393, 11.2%), low, chronic, PFS (45/393, 10.4%), high, acute, PFS (56/393, 14.2%), high, chronic, PFS (54/393, 13.7%).

1b-v) CONCLUSIONS/DISCUSSION in abstract for negative trials

Conclusions/Discussions in abstract for negative trials: Discuss the primary outcome - if the trial is negative (primary outcome not changed), and the intervention was not used, discuss whether negative results are attributable to lack of uptake and discuss reasons. (Note: Only report in the abstract what the main paper is reporting. If this information is missing from the main body of text, consider adding it)

	1	2	3	4	5	
subitem not at all important	○	○	○	○	◉	essential

Sie bearbeiten gerade Ihre Antwort. Wenn Sie die URL freigeben, können andere Personen Ihre Antwort ebenfalls bearbeiten.

NEUE ANTWORT AUSFÜLLEN

Does your paper address subitem 1b-v?

Copy and paste relevant sections from the manuscript abstract (include quotes in quotation marks "like this" to indicate direct quotes from your manuscript), or elaborate on this item by providing additional information not in the ms, or briefly explain why the item is not applicable/relevant for your study

Our results demonstrate that PFSs help to increase EHR uploads by mitigating privacy concerns related to stigmatized diseases. This indicates that a PFS is mainly relevant and effective for users with increased privacy risk perceptions, while they do not hurt other users. Thus, implementing PFSs can increase the likelihood that more patients, even those with increased privacy concerns due to stigmatized diseases, upload their data to the EHR, ultimately increasing health equity.

INTRODUCTION

2a) In INTRODUCTION: Scientific background and explanation of rationale

2a-i) Problem and the type of system/solution

Describe the problem and the type of system/solution that is object of the study: intended as stand-alone intervention vs. incorporated in broader health care program? Intended for a particular patient population? Goals of the intervention, e.g., being more cost-effective to other interventions, replace or complement other solutions? (Note: Details about the intervention are provided in “Methods” under 5)

	1	2	3	4	5	
subitem not at all important	(●)	( )	( )	( )	( )	essential

Auswahl löschen

Sie bearbeiten gerade Ihre Antwort. Wenn Sie die URL freigeben, können andere Personen Ihre Antwort ebenfalls bearbeiten.

NEUE ANTWORT AUSFÜLLEN

Does your paper address subitem 2a-i? *

Copy and paste relevant sections from the manuscript (include quotes in quotation marks "like this" to indicate direct quotes from your manuscript), or elaborate on this item by providing additional information not in the ms, or briefly explain why the item is not applicable/relevant for your study

Not applicable, because we didn't evaluate a system as solution for a specific problem.

2a-ii) Scientific background, rationale: What is known about the (type of) system

Scientific background, rationale: What is known about the (type of) system that is the object of the study (be sure to discuss the use of similar systems for other conditions/ diagnoses, if appropiate), motivation for the study, i.e. what are the reasons for and what is the context for this specific study, from which stakeholder viewpoint is the study performed, potential impact of findings [2]. Briefly justify the choice of the comparator.

	1	2	3	4	5	
subitem not at all important	○	○	○	○	◉	essential

Auswahl löschen

Does your paper address subitem 2a-ii? *

Copy and paste relevant sections from the manuscript (include quotes in quotation marks "like this" to indicate direct quotes from your manuscript), or elaborate on this item by providing additional information not in the ms, or briefly explain why the item is not applicable/relevant for your study

"Disease-specific stigma has been shown to have an inhibiting influence on upload behavior and to increase the risks people perceive when they are asked to upload these diseases to the EHR [11,12]. Conversely, the time course of diseases, i.e., whether diseases are chronic rather than acute, tends to increase both privacy concerns and intention to use the EHR. That is, patients with chronic conditions recognize a greater value in using the EHR but have heightened privacy concerns when it comes to uploading chronic conditions to the EHR [13]. In this study, we aim at merging these two lines of studies to validate and extend the positive effect of a patient-framed PFS on users' decision to upload diseases to the EHR when disease-specific privacy concerns are systematically varied."

Sie bearbeiten gerade Ihre Antwort. Wenn Sie die URL freigeben, können andere Personen Ihre Antwort ebenfalls bearbeiten. NEUE ANTWORT AUSFÜLLEN

2b) In INTRODUCTION: Specific objectives or hypotheses

Does your paper address CONSORT subitem 2b? *

Copy and paste relevant sections from the manuscript (include quotes in quotation marks "like this" to indicate direct quotes from your manuscript), or elaborate on this item by providing additional information not in the ms, or briefly explain why the item is not applicable/relevant for your study

"In this study, we test whether displaying a patient-framed PFS shortly before the decision to upload a medical report must be made increases the likelihood that users upload medical reports to the EHR for diseases that vary along two dimensions, time course and disease-specific stigma. After describing the methods and results, we discuss the implications, reflect on the study's limitations, and conclude with a reflection on the objective of this study."

METHODS

3a) Description of trial design (such as parallel, factorial) including allocation ratio

Does your paper address CONSORT subitem 3a? *

Copy and paste relevant sections from the manuscript (include quotes in quotation marks "like this" to indicate direct quotes from your manuscript), or elaborate on this item by providing additional information not in the ms, or briefly explain why the item is not applicable/relevant for your study

"We used a 2x2x2 between-subject study design with the three independent variables (IVs) stigmatization potential, time course and privacy information. [...] Participants were randomly (single-blinded) assigned to one of the conditions in parallel (simple randomization, ratio: 1:1:1:1:1:1:1:1) using LimeSurvey's built-in “rand” function."

3b) Important changes to methods after trial commencement (such as eligibility

Sie bearbeiten gerade Ihre Antwort. Wenn Sie die URL freigeben, können andere Personen Ihre Antwort ebenfalls bearbeiten. NEUE ANTWORT AUSFÜLLEN

Does your paper address CONSORT subitem 3b? *

Copy and paste relevant sections from the manuscript (include quotes in quotation marks "like this" to indicate direct quotes from your manuscript), or elaborate on this item by providing additional information not in the ms, or briefly explain why the item is not applicable/relevant for your study

We did not make any changes after trial commencement.

3b-i) Bug fixes, Downtimes, Content Changes

Bug fixes, Downtimes, Content Changes: ehealth systems are often dynamic systems. A description of changes to methods therefore also includes important changes made on the intervention or comparator during the trial (e.g., major bug fixes or changes in the functionality or content) (5-iii) and other “unexpected events” that may have influenced study design such as staff changes, system failures/downtimes, etc. [2].

	1	2	3	4	5	
subitem not at all important	◉	○	○	○	○	essential

Auswahl löschen

Does your paper address subitem 3b-i?

Copy and paste relevant sections from the manuscript (include quotes in quotation marks "like this" to indicate direct quotes from your manuscript), or elaborate on this item by providing additional information not in the ms, or briefly explain why the item is not applicable/relevant for your study

There were no bugs or downtimes

4a) Eligibility criteria for participants

Sie bearbeiten gerade Ihre Antwort. Wenn Sie die URL freigeben, können andere Personen Ihre Antwort ebenfalls bearbeiten.

NEUE ANTWORT AUSFÜLLEN

Does your paper address CONSORT subitem 4a? *

Copy and paste relevant sections from the manuscript (include quotes in quotation marks "like this" to indicate direct quotes from your manuscript), or elaborate on this item by providing additional information not in the ms, or briefly explain why the item is not applicable/relevant for your study

"Individuals aged 18 years and older residing in Germany were allowed to participate in the study, as the content and questions of the study were designed to fit the context of the German EHR. Another prerequisite was that participants had no personal previous experience (own illness) with the diseases mentioned in the medical reports we used for this study, as the handling of stigmatized diseases by affected persons is different from that of unaffected persons [33]."

4a-i) Computer / Internet literacy

Computer / Internet literacy is often an implicit “de facto” eligibility criterion - this should be explicitly clarified.

	1	2	3	4	5	
subitem not at all important	(●)	○	○	○	○	essential

Auswahl löschen

Does your paper address subitem 4a-i?

Copy and paste relevant sections from the manuscript (include quotes in quotation marks "like this" to indicate direct quotes from your manuscript), or elaborate on this item by providing additional information not in the ms, or briefly explain why the item is not applicable/relevant for your study

General computer/internet skills did not play a role in our study, as we asked about the more differentiated use of mHealth applications as a control variable.

Sie bearbeiten gerade Ihre Antwort. Wenn Sie die URL freigeben, können andere Personen Ihre Antwort ebenfalls bearbeiten.

NEUE ANTWORT AUSFÜLLEN

4a-ii) Open vs. closed, web-based vs. face-to-face assessments:

Open vs. closed, web-based vs. face-to-face assessments: Mention how participants were recruited (online vs. offline), e.g., from an open access website or from a clinic, and clarify if this was a purely web-based trial, or there were face-to-face components (as part of the intervention or for assessment), i.e., to what degree got the study team to know the participant. In online-only trials, clarify if participants were quasi-anonymous and whether having multiple identities was possible or whether technical or logistical measures (e.g., cookies, email confirmation, phone calls) were used to detect/prevent these.

	1	2	3	4	5	
subitem not at all important	○	○	○	○	◉	essential

Auswahl löschen

Does your paper address subitem 4a-ii? *

Copy and paste relevant sections from the manuscript (include quotes in quotation marks "like this" to indicate direct quotes from your manuscript), or elaborate on this item by providing additional information not in the ms, or briefly explain why the item is not applicable/relevant for your study

"Sampling was conducted through Prolific, a crowdsourcing platform used to recruit participants for online surveys and experiments, known for its diverse participant pool and high data quality [34]."

4a-iii) Information giving during recruitment

Information given during recruitment. Specify how participants were briefed for recruitment and in the informed consent procedures (e.g., publish the informed consent documentation as appendix, see also item X26), as this information may have an effect on user self-selection, user expectation and may also bias results.

	1	2	3	4	5	
subitem not at all important	○	○	○	○	◉	essential

Auswahl löschen

Sie bearbeiten gerade Ihre Antwort. Wenn Sie die URL freigeben, können andere Personen Ihre Antwort ebenfalls bearbeiten.

NEUE ANTWORT AUSFÜLLEN

Does your paper address subitem 4a-iii?

Copy and paste relevant sections from the manuscript (include quotes in quotation marks "like this" to indicate direct quotes from your manuscript), or elaborate on this item by providing additional information not in the ms, or briefly explain why the item is not applicable/relevant for your study

"On the first page of the survey, participants were told about the experimenter, the study purpose, what data were to be collected during the study, and where and for how long they would be stored. Also, participants had the possibility to download a pdf with the study information. Hence, participants were informed about the duration of the survey (approximately 8 minutes) as well as the compensation for participation."

4b) Settings and locations where the data were collected

Does your paper address CONSORT subitem 4b? *

Copy and paste relevant sections from the manuscript (include quotes in quotation marks "like this" to indicate direct quotes from your manuscript), or elaborate on this item by providing additional information not in the ms, or briefly explain why the item is not applicable/relevant for your study

"As in previous studies, we tested the effect of the independent variables by querying the perceived risk and perceived benefit of uploading findings to the EHR using validated items [11–13]. [...] Perceived risk and perceived benefit were measured using a 7-point Likert scale ranging from 1 ("Strongly disagree") to 7 ("Strongly agree"). The decision to upload the finding was measured using a validated dichotomous item (yes/no) [11,12]."

4b-i) Report if outcomes were (self-)assessed through online questionnaires

Clearly report if outcomes were (self-)assessed through online questionnaires (as common in web-based trials) or otherwise.

	1	2	3	4	5	
subitem not at all important	○	○	○	○	◉	essential

Sie bearbeiten gerade Ihre Antwort. Wenn Sie die URL freigeben, können andere Personen Ihre Antwort ebenfalls bearbeiten. **NEUE ANTWORT AUSFÜLLEN**

Does your paper address subitem 4b-i? *

Copy and paste relevant sections from the manuscript (include quotes in quotation marks "like this" to indicate direct quotes from your manuscript), or elaborate on this item by providing additional information not in the ms, or briefly explain why the item is not applicable/relevant for your study

"As in previous studies, we tested the effect of the independent variables by querying the perceived risk and perceived benefit of uploading findings to the EHR using validated items [11–13]. [...] Perceived risk and perceived benefit were measured using a 7-point Likert scale ranging from 1 ("Strongly disagree") to 7 ("Strongly agree"). The decision to upload the finding was measured using a validated dichotomous item (yes/no) [11,12]."

4b-ii) Report how institutional affiliations are displayed

Report how institutional affiliations are displayed to potential participants [on ehealth media], as affiliations with prestigious hospitals or universities may affect volunteer rates, use, and reactions with regards to an intervention.(Not a required item – describe only if this may bias results)

	1	2	3	4	5	
subitem not at all important	○	○	○	○	◉	essential

Auswahl löschen

Does your paper address subitem 4b-ii?

Copy and paste relevant sections from the manuscript (include quotes in quotation marks "like this" to indicate direct quotes from your manuscript), or elaborate on this item by providing additional information not in the ms, or briefly explain why the item is not applicable/relevant for your study

In the study information part, affilations were shown to participants in the contact section.

5) The interventions for each group with sufficient details to allow replication, including how and when they were actually administered

Sie bearbeiten gerade Ihre Antwort. Wenn Sie die URL freigeben, können andere Personen Ihre Antwort ebenfalls bearbeiten.

NEUE ANTWORT AUSFÜLLEN

5-i) Mention names, credential, affiliations of the developers, sponsors, and owners

Mention names, credential, affiliations of the developers, sponsors, and owners [6] (if authors/evaluators are owners or developer of the software, this needs to be declared in a "Conflict of interest" section or mentioned elsewhere in the manuscript).

	1	2	3	4	5	
subitem not at all important	◉	○	○	○	○	essential

Auswahl löschen

Does your paper address subitem 5-i?

Copy and paste relevant sections from the manuscript (include quotes in quotation marks "like this" to indicate direct quotes from your manuscript), or elaborate on this item by providing additional information not in the ms, or briefly explain why the item is not applicable/relevant for your study

The project was funded by the home institution of the authers. No external funding was required.

5-ii) Describe the history/development process

Describe the history/development process of the application and previous formative evaluations (e.g., focus groups, usability testing), as these will have an impact on adoption/use rates and help with interpreting results.

	1	2	3	4	5	
subitem not at all important	○	○	○	○	◉	essential

Auswahl löschen

Sie bearbeiten gerade Ihre Antwort. Wenn Sie die URL freigeben, können andere Personen Ihre Antwort ebenfalls bearbeiten.

NEUE ANTWORT AUSFÜLLEN

Does your paper address subitem 5-ii?

Copy and paste relevant sections from the manuscript (include quotes in quotation marks "like this" to indicate direct quotes from your manuscript), or elaborate on this item by providing additional information not in the ms, or briefly explain why the item is not applicable/relevant for your study

We created the medical findings using MS Word. We created the PFSs using FIGMA. All stimulus material was tested in pilot studies.

5-iii) Revisions and updating

Revisions and updating. Clearly mention the date and/or version number of the application/intervention (and comparator, if applicable) evaluated, or describe whether the intervention underwent major changes during the evaluation process, or whether the development and/or content was “frozen” during the trial. Describe dynamic components such as news feeds or changing content which may have an impact on the replicability of the intervention (for unexpected events see item 3b).

	1	2	3	4	5	
subitem not at all important	(●)	( )	( )	( )	( )	essential

Auswahl löschen

Does your paper address subitem 5-iii?

Copy and paste relevant sections from the manuscript (include quotes in quotation marks "like this" to indicate direct quotes from your manuscript), or elaborate on this item by providing additional information not in the ms, or briefly explain why the item is not applicable/relevant for your study

Nit applicable, because no changes were made.

Sie bearbeiten gerade Ihre Antwort. Wenn Sie die URL freigeben, können andere Personen Ihre Antwort ebenfalls bearbeiten.

NEUE ANTWORT AUSFÜLLEN

5-iv) Quality assurance methods

Provide information on quality assurance methods to ensure accuracy and quality of information provided [1], if applicable.

	1	2	3	4	5	
subitem not at all important	○	○	○	○	◉	essential

Auswahl löschen

Does your paper address subitem 5-iv?

Copy and paste relevant sections from the manuscript (include quotes in quotation marks "like this" to indicate direct quotes from your manuscript), or elaborate on this item by providing additional information not in the ms, or briefly explain why the item is not applicable/relevant for your study

All stimulus material was tested in several pilot studies to ensure high quality.

5-v) Ensure replicability by publishing the source code, and/or providing screenshots/screen-capture video, and/or providing flowcharts of the algorithms used

Ensure replicability by publishing the source code, and/or providing screenshots/screen-capture video, and/or providing flowcharts of the algorithms used. Replicability (i.e., other researchers should in principle be able to replicate the study) is a hallmark of scientific reporting.

	1	2	3	4	5	
subitem not at all important	○	○	○	○	◉	essential

Auswahl löschen

Sie bearbeiten gerade Ihre Antwort. Wenn Sie die URL freigeben, können andere Personen Ihre Antwort ebenfalls bearbeiten.

NEUE ANTWORT AUSFÜLLEN

Does your paper address subitem 5-v?

Copy and paste relevant sections from the manuscript (include quotes in quotation marks "like this" to indicate direct quotes from your manuscript), or elaborate on this item by providing additional information not in the ms, or briefly explain why the item is not applicable/relevant for your study

The used stimuli are attached in the multimedia appendix

5-vi) Digital preservation

Digital preservation: Provide the URL of the application, but as the intervention is likely to change or disappear over the course of the years; also make sure the intervention is archived (Internet Archive, webcitation.org, and/or publishing the source code or screenshots/videos alongside the article). As pages behind login screens cannot be archived, consider creating demo pages which are accessible without login.

	1	2	3	4	5	
subitem not at all important	(●)	( )	( )	( )	( )	essential

Auswahl löschen

Does your paper address subitem 5-vi?

Copy and paste relevant sections from the manuscript (include quotes in quotation marks "like this" to indicate direct quotes from your manuscript), or elaborate on this item by providing additional information not in the ms, or briefly explain why the item is not applicable/relevant for your study

not applicable, because we didn't develop a specific system

Sie bearbeiten gerade Ihre Antwort. Wenn Sie die URL freigeben, können andere Personen Ihre Antwort ebenfalls bearbeiten.

NEUE ANTWORT AUSFÜLLEN

5-vii) Access

Access: Describe how participants accessed the application, in what setting/context, if they had to pay (or were paid) or not, whether they had to be a member of specific group. If known, describe how participants obtained “access to the platform and Internet” [1]. To ensure access for editors/reviewers/readers, consider to provide a “backdoor” login account or demo mode for reviewers/readers to explore the application (also important for archiving purposes, see vi).

	1	2	3	4	5	
subitem not at all important	○	○	○	○	◉	essential

Auswahl löschen

Does your paper address subitem 5-vii? *

Copy and paste relevant sections from the manuscript (include quotes in quotation marks "like this" to indicate direct quotes from your manuscript), or elaborate on this item by providing additional information not in the ms, or briefly explain why the item is not applicable/relevant for your study

The questionnaire was rolled out as a voluntary, open survey that was only accessible via the recruitment platform Prolific.

Sie bearbeiten gerade Ihre Antwort. Wenn Sie die URL freigeben, können andere Personen Ihre Antwort ebenfalls bearbeiten.

NEUE ANTWORT AUSFÜLLEN

5-viii) Mode of delivery, features/functionalities/components of the intervention and comparator, and the theoretical framework

Describe mode of delivery, features/functionalities/components of the intervention and comparator, and the theoretical framework [6] used to design them (instructional strategy [1], behaviour change techniques, persuasive features, etc., see e.g., [7, 8] for terminology). This includes an in-depth description of the content (including where it is coming from and who developed it) [1]," whether [and how] it is tailored to individual circumstances and allows users to track their progress and receive feedback" [6]. This also includes a description of communication delivery channels and – if computer-mediated communication is a component – whether communication was synchronous or asynchronous [6]. It also includes information on presentation strategies [1], including page design principles, average amount of text on pages, presence of hyperlinks to other resources, etc. [1].

	1	2	3	4	5	
subitem not at all important	○	○	○	○	◉	essential

Auswahl löschen

Does your paper address subitem 5-viii? *

Copy and paste relevant sections from the manuscript (include quotes in quotation marks "like this" to indicate direct quotes from your manuscript), or elaborate on this item by providing additional information not in the ms, or briefly explain why the item is not applicable/relevant for your study

Depending on the experimental group, participants were provided with a specific medical finding and a brief description of it (manipulation of stigma and time course) and either a PFS or no privacy information.

Sie bearbeiten gerade Ihre Antwort. Wenn Sie die URL freigeben, können andere Personen Ihre Antwort ebenfalls bearbeiten.

NEUE ANTWORT AUSFÜLLEN

5-ix) Describe use parameters

Describe use parameters (e.g., intended “doses” and optimal timing for use). Clarify what instructions or recommendations were given to the user, e.g., regarding timing, frequency, heaviness of use, if any, or was the intervention used ad libitum.

	1	2	3	4	5	
subitem not at all important	◉	○	○	○	○	essential

Auswahl löschen

Does your paper address subitem 5-ix?

Copy and paste relevant sections from the manuscript (include quotes in quotation marks "like this" to indicate direct quotes from your manuscript), or elaborate on this item by providing additional information not in the ms, or briefly explain why the item is not applicable/relevant for your study

Not applicable, because the stimuli were visual cues (only shown once) and not a medical dose.

5-x) Clarify the level of human involvement

Clarify the level of human involvement (care providers or health professionals, also technical assistance) in the e-intervention or as co-intervention (detail number and expertise of professionals involved, if any, as well as “type of assistance offered, the timing and frequency of the support, how it is initiated, and the medium by which the assistance is delivered”. It may be necessary to distinguish between the level of human involvement required for the trial, and the level of human involvement required for a routine application outside of a RCT setting (discuss under item 21 – generalizability).

	1	2	3	4	5	
subitem not at all important	◉	○	○	○	○	essential

Auswahl löschen

Sie bearbeiten gerade Ihre Antwort. Wenn Sie die URL freigeben, können andere Personen Ihre Antwort ebenfalls bearbeiten.

NEUE ANTWORT AUSFÜLLEN

Does your paper address subitem 5-x?

Copy and paste relevant sections from the manuscript (include quotes in quotation marks "like this" to indicate direct quotes from your manuscript), or elaborate on this item by providing additional information not in the ms, or briefly explain why the item is not applicable/relevant for your study

not applicable, we didn't evaluate a speciic system

5-xi) Report any prompts/reminders used

Report any prompts/reminders used: Clarify if there were prompts (letters, emails, phone calls, SMS) to use the application, what triggered them, frequency etc. It may be necessary to distinguish between the level of prompts/reminders required for the trial, and the level of prompts/reminders for a routine application outside of a RCT setting (discuss under item 21 – generalizability).

	1	2	3	4	5	
subitem not at all important	(x)	( )	( )	( )	( )	essential

Auswahl löschen

Does your paper address subitem 5-xi? *

Copy and paste relevant sections from the manuscript (include quotes in quotation marks "like this" to indicate direct quotes from your manuscript), or elaborate on this item by providing additional information not in the ms, or briefly explain why the item is not applicable/relevant for your study

There were no prompts or reminders

Sie bearbeiten gerade Ihre Antwort. Wenn Sie die URL freigeben, können andere Personen Ihre Antwort ebenfalls bearbeiten.

NEUE ANTWORT AUSFÜLLEN

5-xii) Describe any co-interventions (incl. training/support)

Describe any co-interventions (incl. training/support): Clearly state any interventions that are provided in addition to the targeted eHealth intervention, as ehealth intervention may not be designed as stand-alone intervention. This includes training sessions and support [1]. It may be necessary to distinguish between the level of training required for the trial, and the level of training for a routine application outside of a RCT setting (discuss under item 21 – generalizability.

	1	2	3	4	5	
subitem not at all important	(●)	( )	( )	( )	( )	essential

Auswahl löschen

Does your paper address subitem 5-xii? *

Copy and paste relevant sections from the manuscript (include quotes in quotation marks "like this" to indicate direct quotes from your manuscript), or elaborate on this item by providing additional information not in the ms, or briefly explain why the item is not applicable/relevant for your study

There were no co-interventions

6a) Completely defined pre-specified primary and secondary outcome measures, including how and when they were assessed

Does your paper address CONSORT subitem 6a? *

Copy and paste relevant sections from the manuscript (include quotes in quotation marks "like this" to indicate direct quotes from your manuscript), or elaborate on this item by providing additional information not in the ms, or briefly explain why the item is not applicable/relevant for your study

The uploading behavior (primary outcome, measured using a dichotomous yes/no item) and the perceived benefits and risks (secondory outcome as manipulation check, item 7 point likert scale) were measured after the interaction with the medical finding and the interaction

Sie bearbeiten gerade Ihre Antwort. Wenn Sie die URL freigeben, können andere Personen Ihre Antwort ebenfalls bearbeiten.

NEUE ANTWORT AUSFÜLLEN

6a-i) Online questionnaires: describe if they were validated for online use and apply CHERRIES items to describe how the questionnaires were designed/deployed

If outcomes were obtained through online questionnaires, describe if they were validated for online use and apply CHERRIES items to describe how the questionnaires were designed/deployed [9].

	1	2	3	4	5	
subitem not at all important	○	○	○	○	◉	essential

Auswahl löschen

Does your paper address subitem 6a-i?

Copy and paste relevant sections from manuscript text

We used the validated measurements of "von Kalckreuth, N., & Feufel, M. A. (2024). Influence of Disease-Related Stigma on Patients' Decisions to Upload Medical Reports to the German Electronic Health Record: Randomized Controlled Trial. JMIR Human Factors, 11, e52625. https://doi.org/10.2196/52625" and "von Kalckreuth, N., Prümper, A. M., & Feufel, M. A. (2023). The Influence of Health Data on the Use of the Electronic Health Record (EHR) – a Mixed Methods Approach. AMCIS 2023 Proceedings, 2. https://aisel.aisnet.org/amcis2023/sig_health/sig_health/2"

6a-ii) Describe whether and how "use" (including intensity of use/dosage) was defined/measured/monitored

Describe whether and how "use" (including intensity of use/dosage) was defined/measured/monitored (logins, logfile analysis, etc.). Use/adoption metrics are important process outcomes that should be reported in any ehealth trial.

	1	2	3	4	5	
subitem not at all important	◉	○	○	○	○	essential

Auswahl löschen

Sie bearbeiten gerade Ihre Antwort. Wenn Sie die URL freigeben, können andere Personen Ihre Antwort ebenfalls bearbeiten.

NEUE ANTWORT AUSFÜLLEN

Does your paper address subitem 6a-ii?

Copy and paste relevant sections from manuscript text

Not applicable, because participants were shown the medical findings and privacy information automatically.

6a-iii) Describe whether, how, and when qualitative feedback from participants was obtained

Describe whether, how, and when qualitative feedback from participants was obtained (e.g., through emails, feedback forms, interviews, focus groups).

	1	2	3	4	5	
subitem not at all important	○	◉	○	○	○	essential

Auswahl löschen

Does your paper address subitem 6a-iii?

Copy and paste relevant sections from manuscript text

Our study included an open text field where participants could enter any questions or feedback. Additionally, they had the option to send messages through their Prolific account. However, we did not include qualitative data in our analysis.

6b) Any changes to trial outcomes after the trial commenced, with reasons

Sie bearbeiten gerade Ihre Antwort. Wenn Sie die URL freigeben, können andere Personen Ihre Antwort ebenfalls bearbeiten.

NEUE ANTWORT AUSFÜLLEN

Does your paper address CONSORT subitem 6b? *

Copy and paste relevant sections from the manuscript (include quotes in quotation marks "like this" to indicate direct quotes from your manuscript), or elaborate on this item by providing additional information not in the ms, or briefly explain why the item is not applicable/relevant for your study

We did not make any changes after trial commencement.

7a) How sample size was determined

NPT: When applicable, details of whether and how the clustering by care provides or centers was addressed

7a-i) Describe whether and how expected attrition was taken into account when calculating the sample size

Describe whether and how expected attrition was taken into account when calculating the sample size.

	1	2	3	4	5	
subitem not at all important	○	○	○	○	◉	essential

Auswahl löschen

Sie bearbeiten gerade Ihre Antwort. Wenn Sie die URL freigeben, können andere Personen Ihre Antwort ebenfalls bearbeiten.

NEUE ANTWORT AUSFÜLLEN

Does your paper address subitem 7a-i?

Copy and paste relevant sections from manuscript title (include quotes in quotation marks "like this" to indicate direct quotes from your manuscript), or elaborate on this item by providing additional information not in the ms, or briefly explain why the item is not applicable/relevant for your study

"Based on an a priori power analysis for a logistic regression using G*Power (version 3.1.9.7) with disease-related stigma (high vs low), time course (acute vs chronic) and privacy information (PFS vs no PFS) as binomial distributed predictors, a false positive rate α=0.05, a power of β=0.80, an estimated odds ratio for the predictor with the smallest expected effect size (time course) OR 1.7 (derived from the pre study with n=80 participants), and the probability of the outcome (upload decision) under the null hypothesis of 0.5, reflecting a conservative assumption, we aimed for a sample size of n=363 participants. To ensure this target was met, we oversampled participants by 30%, resulting in a total sample of 471 individuals. Oversampling accounted for potential exclusions due to failed attention checks, study dropouts, self-reported invalid data (approximately 20%, as indicated in preliminary studies[31,32], and prior medical histories with the diseases used in the study (approximately 10%, based on prior findings) [11]."

7b) When applicable, explanation of any interim analyses and stopping guidelines

Does your paper address CONSORT subitem 7b? *

Copy and paste relevant sections from the manuscript (include quotes in quotation marks "like this" to indicate direct quotes from your manuscript), or elaborate on this item by providing additional information not in the ms, or briefly explain why the item is not applicable/relevant for your study

We stopped data collection once we reached our desired sample size.

8a) Method used to generate the random allocation sequence

NPT: When applicable, how care providers were allocated to each trial group

Sie bearbeiten gerade Ihre Antwort. Wenn Sie die URL freigeben, können andere Personen Ihre Antwort ebenfalls bearbeiten.

NEUE ANTWORT AUSFÜLLEN

Does your paper address CONSORT subitem 8a? *

Copy and paste relevant sections from the manuscript (include quotes in quotation marks "like this" to indicate direct quotes from your manuscript), or elaborate on this item by providing additional information not in the ms, or briefly explain why the item is not applicable/relevant for your study

"Participants were randomly (single-blinded) assigned to one of the conditions in parallel (simple randomization, ratio: 1:1:1:1:1:1:1:1) using LimeSurvey's built-in "rand" function."

8b) Type of randomisation; details of any restriction (such as blocking and block size)

Does your paper address CONSORT subitem 8b? *

Copy and paste relevant sections from the manuscript (include quotes in quotation marks "like this" to indicate direct quotes from your manuscript), or elaborate on this item by providing additional information not in the ms, or briefly explain why the item is not applicable/relevant for your study

"Participants were randomly (single-blinded) assigned to one of the conditions in parallel (simple randomization, ratio: 1:1:1:1:1:1:1:1) using LimeSurvey's built-in "rand" function."

9) Mechanism used to implement the random allocation sequence (such as sequentially numbered containers), describing any steps taken to conceal the sequence until interventions were assigned

Does your paper address CONSORT subitem 9? *

Copy and paste relevant sections from the manuscript (include quotes in quotation marks "like this" to indicate direct quotes from your manuscript), or elaborate on this item by providing additional information not in the ms, or briefly explain why the item is not applicable/relevant for your study

"Participants were randomly (single-blinded) assigned to one of the conditions in parallel

Sie bearbeiten gerade Ihre Antwort. Wenn Sie die URL freigeben, können andere Personen Ihre Antwort ebenfalls bearbeiten.

NEUE ANTWORT AUSFÜLLEN

10) Who generated the random allocation sequence, who enrolled participants, and who assigned participants to interventions

Does your paper address CONSORT subitem 10? *

Copy and paste relevant sections from the manuscript (include quotes in quotation marks "like this" to indicate direct quotes from your manuscript), or elaborate on this item by providing additional information not in the ms, or briefly explain why the item is not applicable/relevant for your study

"Participants were randomly (single-blinded) assigned to one of the conditions in parallel (simple randomization, ratio: 1:1:1:1:1:1:1:1) using LimeSurvey's built-in “rand” function." The questionnaire was rolled out as a voluntary, open survey that was only accessible via the recruitment platform prolific.

11a) If done, who was blinded after assignment to interventions (for example, participants, care providers, those assessing outcomes) and how

NPT: Whether or not administering co-interventions were blinded to group assignment

11a-i) Specify who was blinded, and who wasn’t

Specify who was blinded, and who wasn’t. Usually, in web-based trials it is not possible to blind the participants [1, 3] (this should be clearly acknowledged), but it may be possible to blind outcome assessors, those doing data analysis or those administering co-interventions (if any).

	1	2	3	4	5	
subitem not at all important	○	○	○	○	◉	essential

Auswahl löschen

Sie bearbeiten gerade Ihre Antwort. Wenn Sie die URL freigeben, können andere Personen Ihre Antwort ebenfalls bearbeiten.

NEUE ANTWORT AUSFÜLLEN

Does your paper address subitem 11a-i? *

Copy and paste relevant sections from the manuscript (include quotes in quotation marks "like this" to indicate direct quotes from your manuscript), or elaborate on this item by providing additional information not in the ms, or briefly explain why the item is not applicable/relevant for your study

Participants could not be blinded in the study by design, but they were not aware, that other framing conditions exist while taking part in the study.

11a-ii) Discuss e.g., whether participants knew which intervention was the "intervention of interest" and which one was the "comparator"

Informed consent procedures (4a-ii) can create biases and certain expectations - discuss e.g., whether participants knew which intervention was the "intervention of interest" and which one was the "comparator".

	1	2	3	4	5	
subitem not at all important	(●)	○	○	○	○	essential

Auswahl löschen

Does your paper address subitem 11a-ii?

Copy and paste relevant sections from the manuscript (include quotes in quotation marks "like this" to indicate direct quotes from your manuscript), or elaborate on this item by providing additional information not in the ms, or briefly explain why the item is not applicable/relevant for your study

Not applicable. All participants just had one intervention. A comparison of "Intervention of interest" was not possible.

11b) If relevant, description of the similarity of interventions

(this item is usually not relevant for ehealth trials as it refers to similarity of a placebo or sham intervention to a active medication/intervention)

Sie bearbeiten gerade Ihre Antwort. Wenn Sie die URL freigeben, können andere Personen Ihre Antwort ebenfalls bearbeiten. NEUE ANTWORT AUSFÜLLEN

Does your paper address CONSORT subitem 11b? *

Copy and paste relevant sections from the manuscript (include quotes in quotation marks "like this" to indicate direct quotes from your manuscript), or elaborate on this item by providing additional information not in the ms, or briefly explain why the item is not applicable/relevant for your study

Not applicable, as we did not employ placebo.

12a) Statistical methods used to compare groups for primary and secondary outcomes

NPT: When applicable, details of whether and how the clustering by care providers or centers was addressed

Does your paper address CONSORT subitem 12a? *

Copy and paste relevant sections from the manuscript (include quotes in quotation marks "like this" to indicate direct quotes from your manuscript), or elaborate on this item by providing additional information not in the ms, or briefly explain why the item is not applicable/relevant for your study

"The analyses regarding the manipulation checks of perceived privacy risks and benefits were performed using t-tests , a statistical method used to compare the means of two groups. The influence of the IVs (disease-specific stigma, time course, displaying a PFS) and the interaction effects between stigma and the display of a PFS, as well as between time course and the display of a PFS on the upload decision were tested using multiple logistic regression with dummy coding , a method used to model the probability of a binary outcome based on one or more predictor variables."

Sie bearbeiten gerade Ihre Antwort. Wenn Sie die URL freigeben, können andere Personen Ihre Antwort ebenfalls bearbeiten.

NEUE ANTWORT AUSFÜLLEN

12a-i) Imputation techniques to deal with attrition / missing values

Imputation techniques to deal with attrition / missing values: Not all participants will use the intervention/comparator as intended and attrition is typically high in ehealth trials. Specify how participants who did not use the application or dropped out from the trial were treated in the statistical analysis (a complete case analysis is strongly discouraged, and simple imputation techniques such as LOCF may also be problematic [4]).

	1	2	3	4	5	
subitem not at all important	(●)	( )	( )	( )	( )	essential

Auswahl löschen

Does your paper address subitem 12a-i? *

Copy and paste relevant sections from the manuscript (include quotes in quotation marks "like this" to indicate direct quotes from your manuscript), or elaborate on this item by providing additional information not in the ms, or briefly explain why the item is not applicable/relevant for your study

Participants that did not finish the questionnaire were excluded from the analysis. Thus we didn't have to handle missing data.

12b) Methods for additional analyses, such as subgroup analyses and adjusted analyses

Sie bearbeiten gerade Ihre Antwort. Wenn Sie die URL freigeben, können andere Personen Ihre Antwort ebenfalls bearbeiten.

NEUE ANTWORT AUSFÜLLEN

Does your paper address CONSORT subitem 12b? *

Copy and paste relevant sections from the manuscript (include quotes in quotation marks "like this" to indicate direct quotes from your manuscript), or elaborate on this item by providing additional information not in the ms, or briefly explain why the item is not applicable/relevant for your study

"We also included a robustness check of the results regarding the upload decision. To control for potential influences of demographic and interindividual variables that could bias coefficients and P values, we used multiple logistic regression. To not bias P values as a result of controlling, we only included variables in the model that have been shown to have a causal relationship with the independent variables (i.e., causal confounders): age, education level, and experience with the technical system [43–45]. P values were adjusted for multiple testing using the Benjamini-Hochberg procedure [46]."

X26) REB/IRB Approval and Ethical Considerations [recommended as subheading under "Methods"] (not a CONSORT item)

X26-i) Comment on ethics committee approval

	1	2	3	4	5	
subitem not at all important	○	○	○	○	◉	essential

Auswahl löschen

Does your paper address subitem X26-i?

Copy and paste relevant sections from the manuscript (include quotes in quotation marks "like this" to indicate direct quotes from your manuscript), or elaborate on this item by providing additional information not in the ms, or briefly explain why the item is not applicable/relevant for your study

"This study was approved by the Ethics Committee of the Department of Psychology and Ergonomics (IPA) at Technische Universität Berlin (tracking number: AWB_KAL_1_230206_Erweiterungsantrag)."

Sie bearbeiten gerade Ihre Antwort. Wenn Sie die URL freigeben, können andere Personen Ihre Antwort ebenfalls bearbeiten.

NEUE ANTWORT AUSFÜLLEN

x26-ii) Outline informed consent procedures

Outline informed consent procedures e.g., if consent was obtained offline or online (how? Checkbox, etc.?), and what information was provided (see 4a-ii). See [6] for some items to be included in informed consent documents.

	1	2	3	4	5	
subitem not at all important	○	○	○	○	◉	essential

Auswahl löschen

Does your paper address subitem X26-ii?

Copy and paste relevant sections from the manuscript (include quotes in quotation marks "like this" to indicate direct quotes from your manuscript), or elaborate on this item by providing additional information not in the ms, or briefly explain why the item is not applicable/relevant for your study

"Participants volunteered to participate in the survey, and written informed consent was required to participate. "

X26-iii) Safety and security procedures

Safety and security procedures, incl. privacy considerations, and any steps taken to reduce the likelihood or detection of harm (e.g., education and training, availability of a hotline)

	1	2	3	4	5	
subitem not at all important	◉	○	○	○	○	essential

Auswahl löschen

Sie bearbeiten gerade Ihre Antwort. Wenn Sie die URL freigeben, können andere Personen Ihre Antwort ebenfalls bearbeiten.

NEUE ANTWORT AUSFÜLLEN

Does your paper address subitem X26-iii?

Copy and paste relevant sections from the manuscript (include quotes in quotation marks "like this" to indicate direct quotes from your manuscript), or elaborate on this item by providing additional information not in the ms, or briefly explain why the item is not applicable/relevant for your study

Not applicable, because we did not collect any identifying data.

RESULTS

13a) For each group, the numbers of participants who were randomly assigned, received intended treatment, and were analysed for the primary outcome

NPT: The number of care providers or centers performing the intervention in each group and the number of patients treated by each care provider in each center

Does your paper address CONSORT subitem 13a? *

Copy and paste relevant sections from the manuscript (include quotes in quotation marks "like this" to indicate direct quotes from your manuscript), or elaborate on this item by providing additional information not in the ms, or briefly explain why the item is not applicable/relevant for your study

Group 1 (low stigma, acute, no PFS): 53; Group 2 (low stigma, chronic, no PFS): 45; Group 3 (high stigma, acute, no PFS): 46; Group 4 (high stigma, chronic, no PFS): 55; Group 5 (low stigma, acute, PFS): 46; Group 6 (low stigma, chronic, PFS): 42; Group 7 (high stigma, acute, PFS): 58; Group 8 (high stigma, chronic, PFS): 56

13b) For each group, losses and exclusions after randomisation, together with reasons

Sie bearbeiten gerade Ihre Antwort. Wenn Sie die URL freigeben, können andere Personen Ihre Antwort ebenfalls bearbeiten.

NEUE ANTWORT AUSFÜLLEN

Does your paper address CONSORT subitem 13b? (NOTE: Preferably, this is shown in a CONSORT flow diagram) *

Copy and paste relevant sections from the manuscript (include quotes in quotation marks "like this" to indicate direct quotes from your manuscript), or elaborate on this item by providing additional information not in the ms, or briefly explain why the item is not applicable/relevant for your study

Group 1: 1 excluded due self-labeling as invalid; Group 5: 1 excluded due poor attention check, 1 excluded due self-labeling as invalid; Group 6: 1 excluded due poor attention check; Group 7: 1 excluded due poor attention check, 1 excluded due self-labeling as invalid; Group 8: 1 excluded due poor attention check, 1 excluded due self-labeling as invalid;

13b-i) Attrition diagram

Strongly recommended: An attrition diagram (e.g., proportion of participants still logging in or using the intervention/comparator in each group plotted over time, similar to a survival curve) or other figures or tables demonstrating usage/dose/engagement.

	1	2	3	4	5	
subitem not at all important	(●)	( )	( )	( )	( )	essential

Auswahl löschen

Does your paper address subitem 13b-i?

Copy and paste relevant sections from the manuscript or cite the figure number if applicable (include quotes in quotation marks "like this" to indicate direct quotes from your manuscript), or elaborate on this item by providing additional information not in the ms, or briefly explain why the item is not applicable/relevant for your study

Not applicable, since the stimulus material was only presented once.

14a) Dates defining the periods of recruitment and follow-up

Sie bearbeiten gerade Ihre Antwort. Wenn Sie die URL freigeben, können andere Personen Ihre Antwort ebenfalls bearbeiten.

NEUE ANTWORT AUSFÜLLEN

Does your paper address CONSORT subitem 14a? *

Copy and paste relevant sections from the manuscript (include quotes in quotation marks "like this" to indicate direct quotes from your manuscript), or elaborate on this item by providing additional information not in the ms, or briefly explain why the item is not applicable/relevant for your study

"The online study was conducted between April 15, 2024 and May 16, 2024."

14a-i) Indicate if critical “secular events” fell into the study period

Indicate if critical “secular events” fell into the study period, e.g., significant changes in Internet resources available or “changes in computer hardware or Internet delivery resources”

	1	2	3	4	5	
subitem not at all important	(●)	○	○	○	○	essential

Auswahl löschen

Does your paper address subitem 14a-i?

Copy and paste relevant sections from the manuscript (include quotes in quotation marks "like this" to indicate direct quotes from your manuscript), or elaborate on this item by providing additional information not in the ms, or briefly explain why the item is not applicable/relevant for your study

There were no secular events.

14b) Why the trial ended or was stopped (early)

Sie bearbeiten gerade Ihre Antwort. Wenn Sie die URL freigeben, können andere Personen Ihre Antwort ebenfalls bearbeiten.

NEUE ANTWORT AUSFÜLLEN

Does your paper address CONSORT subitem 14b? *

Copy and paste relevant sections from the manuscript (include quotes in quotation marks "like this" to indicate direct quotes from your manuscript), or elaborate on this item by providing additional information not in the ms, or briefly explain why the item is not applicable/relevant for your study

The trial ended once we reached our desired sample size.

15) A table showing baseline demographic and clinical characteristics for each group

NPT: When applicable, a description of care providers (case volume, qualification, expertise, etc.) and centers (volume) in each group

Does your paper address CONSORT subitem 15? *

Copy and paste relevant sections from the manuscript (include quotes in quotation marks "like this" to indicate direct quotes from your manuscript), or elaborate on this item by providing additional information not in the ms, or briefly explain why the item is not applicable/relevant for your study

Included in the partitipant section in the results and a complete table in the multimedia appendix.

15-i) Report demographics associated with digital divide issues

In ehealth trials it is particularly important to report demographics associated with digital divide issues, such as age, education, gender, social-economic status, computer/Internet/ehealth literacy of the participants, if known.

	1	2	3	4	5	
subitem not at all important	○	○	○	○	◉	essential

Auswahl löschen

Sie bearbeiten gerade Ihre Antwort. Wenn Sie die URL freigeben, können andere Personen Ihre Antwort ebenfalls bearbeiten.

NEUE ANTWORT AUSFÜLLEN

Does your paper address subitem 15-i? *

Copy and paste relevant sections from the manuscript (include quotes in quotation marks "like this" to indicate direct quotes from your manuscript), or elaborate on this item by providing additional information not in the ms, or briefly explain why the item is not applicable/relevant for your study

Demographics such as age, gender, education and experience with mHealth applications are reported in the participant characteristics.

16) For each group, number of participants (denominator) included in each analysis and whether the analysis was by original assigned groups

16-i) Report multiple “denominators” and provide definitions

Report multiple “denominators” and provide definitions: Report N’s (and effect sizes) “across a range of study participation [and use] thresholds” [1], e.g., N exposed, N consented, N used more than x times, N used more than y weeks, N participants “used” the intervention/comparator at specific pre-defined time points of interest (in absolute and relative numbers per group). Always clearly define “use” of the intervention.

	1	2	3	4	5	
subitem not at all important	○	○	○	○	◉	essential

Auswahl löschen

Does your paper address subitem 16-i? *

Copy and paste relevant sections from the manuscript (include quotes in quotation marks "like this" to indicate direct quotes from your manuscript), or elaborate on this item by providing additional information not in the ms, or briefly explain why the item is not applicable/relevant for your study

All participants were analyzed in their originally assigned groups. Group 1: 52; Group 2: 45; Group 3: 46; Group 4: 55; Group 5: 44; Group 6: 41; Group 7: 56; Group 8: 54.

Sie bearbeiten gerade Ihre Antwort. Wenn Sie die URL freigeben, können andere Personen Ihre Antwort ebenfalls bearbeiten.

NEUE ANTWORT AUSFÜLLEN

16-ii) Primary analysis should be intent-to-treat

Primary analysis should be intent-to-treat, secondary analyses could include comparing only "users", with the appropriate caveats that this is no longer a randomized sample (see 18-i).

	1	2	3	4	5	
subitem not at all important	◉	○	○	○	○	essential

Auswahl löschen

Does your paper address subitem 16-ii?

Copy and paste relevant sections from the manuscript (include quotes in quotation marks "like this" to indicate direct quotes from your manuscript), or elaborate on this item by providing additional information not in the ms, or briefly explain why the item is not applicable/relevant for your study

"The analyses regarding the manipulation checks of perceived privacy risks and benefits were performed using t-tests , a statistical method used to compare the means of two groups. The influence of the IVs (disease-specific stigma, time course, displaying a PFS) and the interaction effects between stigma and the display of a PFS, as well as between time course and the display of a PFS on the upload decision were tested using multiple logistic regression with dummy coding , a method used to model the probability of a binary outcome based on one or more predictor variables.
We also included a robustness check of the results regarding the upload decision. To control for potential influences of demographic and interindividual variables that could bias coefficients and P values, we used multiple logistic regression. To not bias P values as a result of controlling, we only included variables in the model that have been shown to have a causal relationship with the independent variables (i.e., causal confounders): age, education level, and experience with the technical system [43–45]. P values were adjusted for multiple testing using the Benjamini-Hochberg procedure."

17a) For each primary and secondary outcome, results for each group, and the estimated effect size and its precision (such as 95% confidence interval)

Sie bearbeiten gerade Ihre Antwort. Wenn Sie die URL freigeben, können andere Personen Ihre Antwort ebenfalls bearbeiten.

NEUE ANTWORT AUSFÜLLEN

Does your paper address CONSORT subitem 17a? *

Copy and paste relevant sections from the manuscript (include quotes in quotation marks "like this" to indicate direct quotes from your manuscript), or elaborate on this item by providing additional information not in the ms, or briefly explain why the item is not applicable/relevant for your study

"Similar to the preliminary studies [11,12], risk and benefit perception of uploading served as a manipulation check to test the validity of our manipulation (i.e., the medical reports) with respect to the perception of risk (stigma) and benefit (time course). As expected, uploading medical findings of stigmatized diseases was perceived as riskier than those of non-stigmatized diseases (low: mean 3.88, SD 1.68; high: mean 5.15, SD 1.6; t_{391}=7.648; P<.001). Consequently, we assume that our risk manipulation was successful. There was no significant difference of the perceived benefit regarding the time course of the disease (acute: mean 5.63, SD 1.41; chronic: mean 5.72, SD 1.16; t_{391}=0.703, P=.483). Consequently, we assume that our benefit manipulation was not successful.
Additionally, we analyzed the benefit perception in relation to stigma and the risk perception in relation to time course, even though these were not part of the initial manipulation checks. Uploading medical findings of non-stigmatized diseases was perceived as more beneficial than those of stigmatized diseases (low: mean 5.84, SD 1.16; high: mean 5.54, SD 1.39; t_{391}=2.345, P=.019). Furthermore, uploading medical findings of chronic diseases into the EHR was perceived as riskier than those of acute diseases (acute: mean 4.31, SD 1.83; chronic: mean 4.82, SD 1.64; t_{391}=2.893, P=.004)."; "Upload behavior was negatively associated with disease-related stigma (z=4.568, P<.001), thus supporting H1. Specifically, when stigma was high, it was more than seven times less likely that the report was uploaded to the EHR (76.3%; 161/211) than when stigma was low (91.8%; 167/182). Time course of the disease was not associated with the decision to upload a report (z=0.877, P=.380). Consequently, H2 is rejected. The PFS was positively associated with the decision to upload a medical report to the EHR (z=3.298, P<.001), supporting H3. When a PFS was given, participants were more than four times as likely to upload the diagnosis to their EHR (89.2%; 174/195) than when a PFS was not given (77.7%; 154/198)."; "We also tested for interaction effects between stigma and privacy information, as well as between time course and privacy information, to explore potential moderating effects. The interaction between stigma and privacy information was significant (z=2.734, P=.006), indicating that the increase in the number of uploads when showing a PFS is higher for stigmatized diseases than for non-stigmatized diseases, thus supporting H4 (see Figure 6A). In contrast, the interaction between time course and privacy information was not significant (z=0.094, P=.924), suggesting that displaying a PFS did not differentially impact the upload decision based on whether the disease was acute or chronic (see Figure 6B)."

Sie bearbeiten gerade Ihre Antwort. Wenn Sie die URL freigeben, können andere Personen Ihre Antwort ebenfalls bearbeiten.

NEUE ANTWORT AUSFÜLLEN

17a-i) Presentation of process outcomes such as metrics of use and intensity of use

In addition to primary/secondary (clinical) outcomes, the presentation of process outcomes such as metrics of use and intensity of use (dose, exposure) and their operational definitions is critical. This does not only refer to metrics of attrition (13-b) (often a binary variable), but also to more continuous exposure metrics such as “average session length”. These must be accompanied by a technical description how a metric like a “session” is defined (e.g., timeout after idle time) [1] (report under item 6a).

	1	2	3	4	5	
subitem not at all important	○	○	○	○	○	essential

Does your paper address subitem 17a-i?

Copy and paste relevant sections from the manuscript (include quotes in quotation marks "like this" to indicate direct quotes from your manuscript), or elaborate on this item by providing additional information not in the ms, or briefly explain why the item is not applicable/relevant for your study

Not applicable, sinde the intervention was only presented once.

17b) For binary outcomes, presentation of both absolute and relative effect sizes is recommended

Sie bearbeiten gerade Ihre Antwort. Wenn Sie die URL freigeben, können andere Personen Ihre Antwort ebenfalls bearbeiten.

NEUE ANTWORT AUSFÜLLEN

Does your paper address CONSORT subitem 17b? *

Copy and paste relevant sections from the manuscript (include quotes in quotation marks "like this" to indicate direct quotes from your manuscript), or elaborate on this item by providing additional information not in the ms, or briefly explain why the item is not applicable/relevant for your study

"Upload behavior was negatively associated with disease-related stigma (z=4.568, P<.001), thus supporting H1. Specifically, when stigma was high, it was more than seven times less likely that the report was uploaded to the EHR (76.3%; 161/211) than when stigma was low (91.8%; 167/182). Time course of the disease was not associated with the decision to upload a report (z=0.877, P=.380). Consequently, H2 is rejected. The PFS was positively associated with the decision to upload a medical report to the EHR (z=3.298, P<.001), supporting H3. When a PFS was given, participants were more than four times as likely to upload the diagnosis to their EHR (89.2%; 174/195) than when a PFS was not given (77.7%; 154/198)."

18) Results of any other analyses performed, including subgroup analyses and adjusted analyses, distinguishing pre-specified from exploratory

Does your paper address CONSORT subitem 18? *

Copy and paste relevant sections from the manuscript (include quotes in quotation marks "like this" to indicate direct quotes from your manuscript), or elaborate on this item by providing additional information not in the ms, or briefly explain why the item is not applicable/relevant for your study

"We also tested for interaction effects between stigma and privacy information, as well as between time course and privacy information, to explore potential moderating effects. The interaction between stigma and privacy information was significant (z=2.734, P=.006), indicating that the increase in the number of uploads when showing a PFS is higher for stigmatized diseases than for non-stigmatized diseases, thus supporting H4 (see Figure 6A). In contrast, the interaction between time course and privacy information was not significant (z=0.094, P=.924), suggesting that displaying a PFS did not differentially impact the upload decision based on whether the disease was acute or chronic (see Figure 6B)."

Sie bearbeiten gerade Ihre Antwort. Wenn Sie die URL freigeben, können andere Personen Ihre Antwort ebenfalls bearbeiten. NEUE ANTWORT AUSFÜLLEN

18-i) Subgroup analysis of comparing only users

A subgroup analysis of comparing only users is not uncommon in ehealth trials, but if done, it must be stressed that this is a self-selected sample and no longer an unbiased sample from a randomized trial (see 16-iii).

	1	2	3	4	5	
subitem not at all important	◉	○	○	○	○	essential

Auswahl löschen

Does your paper address subitem 18-i?

Copy and paste relevant sections from the manuscript (include quotes in quotation marks "like this" to indicate direct quotes from your manuscript), or elaborate on this item by providing additional information not in the ms, or briefly explain why the item is not applicable/relevant for your study

Not applicable, because we did not conduct subgroup analysis.

19) All important harms or unintended effects in each group
(for specific guidance see CONSORT for harms)

Does your paper address CONSORT subitem 19? *

Copy and paste relevant sections from the manuscript (include quotes in quotation marks "like this" to indicate direct quotes from your manuscript), or elaborate on this item by providing additional information not in the ms, or briefly explain why the item is not applicable/relevant for your study

We did not observe any harms or unintended effects.

Sie bearbeiten gerade Ihre Antwort. Wenn Sie die URL freigeben, können andere Personen Ihre Antwort ebenfalls bearbeiten.

NEUE ANTWORT AUSFÜLLEN

19-i) Include privacy breaches, technical problems

Include privacy breaches, technical problems. This does not only include physical “harm” to participants, but also incidents such as perceived or real privacy breaches [1], technical problems, and other unexpected/unintended incidents. “Unintended effects” also includes unintended positive effects [2].

	1	2	3	4	5	
subitem not at all important	(●)	○	○	○	○	essential

Auswahl löschen

Does your paper address subitem 19-i?

Copy and paste relevant sections from the manuscript (include quotes in quotation marks "like this" to indicate direct quotes from your manuscript), or elaborate on this item by providing additional information not in the ms, or briefly explain why the item is not applicable/relevant for your study

There were no technical problems.

19-ii) Include qualitative feedback from participants or observations from staff/researchers

Include qualitative feedback from participants or observations from staff/researchers, if available, on strengths and shortcomings of the application, especially if they point to unintended/unexpected effects or uses. This includes (if available) reasons for why people did or did not use the application as intended by the developers.

	1	2	3	4	5	
subitem not at all important	(●)	○	○	○	○	essential

Auswahl löschen

Sie bearbeiten gerade Ihre Antwort. Wenn Sie die URL freigeben, können andere Personen Ihre Antwort ebenfalls bearbeiten.

NEUE ANTWORT AUSFÜLLEN

Does your paper address subitem 19-ii?

Copy and paste relevant sections from the manuscript (include quotes in quotation marks "like this" to indicate direct quotes from your manuscript), or elaborate on this item by providing additional information not in the ms, or briefly explain why the item is not applicable/relevant for your study

Since this was an online study using a questionnaire tool (lime survey), we could not collect observation data regarding usage.

DISCUSSION

22) Interpretation consistent with results, balancing benefits and harms, and considering other relevant evidence

NPT: In addition, take into account the choice of the comparator, lack of or partial blinding, and unequal expertise of care providers or centers in each group

22-i) Restate study questions and summarize the answers suggested by the data, starting with primary outcomes and process outcomes (use)

Restate study questions and summarize the answers suggested by the data, starting with primary outcomes and process outcomes (use).

	1	2	3	4	5	
subitem not at all important	○	○	○	○	◉	essential

Auswahl löschen

Sie bearbeiten gerade Ihre Antwort. Wenn Sie die URL freigeben, können andere Personen Ihre Antwort ebenfalls bearbeiten.

NEUE ANTWORT AUSFÜLLEN

Does your paper address subitem 22-i? *

Copy and paste relevant sections from the manuscript (include quotes in quotation marks "like this" to indicate direct quotes from your manuscript), or elaborate on this item by providing additional information not in the ms, or briefly explain why the item is not applicable/relevant for your study

"Displaying a PFS did not influence the decision to upload medical findings for non-stigmatized diseases, as nearly all non-stigmatized medical reports were uploaded regardless of whether a PFS was given (see Figure 6A). In contrast, for stigmatized diseases, the PFS significantly increased the likelihood of uploads (see Figure 6B). This suggests that showing a PFS shortly before a decision to upload medical findings to the EHR must be made is not only effective in mitigating general privacy concerns but helps to reduce specific fears related to stigmatized diseases and increase upload decisions [28,29,31]."

22-ii) Highlight unanswered new questions, suggest future research

Highlight unanswered new questions, suggest future research.

	1	2	3	4	5	
subitem not at all important	○	○	○	○	◉	essential

Auswahl löschen

Sie bearbeiten gerade Ihre Antwort. Wenn Sie die URL freigeben, können andere Personen Ihre Antwort ebenfalls bearbeiten.

NEUE ANTWORT AUSFÜLLEN

Does your paper address subitem 22-ii?

Copy and paste relevant sections from the manuscript (include quotes in quotation marks "like this" to indicate direct quotes from your manuscript), or elaborate on this item by providing additional information not in the ms, or briefly explain why the item is not applicable/relevant for your study

"It is clear that the adoption and approval of data-gathering technologies are strongly influenced by cultural differences [52]. In comparison to other European nations, the German population exhibits a heightened level of caution regarding the use of personal information online [53]. Given that in this study data collection was conducted solely with residents of Germany, future studies should validate the applicability of these findings in other countries. We deliberately excluded participants who already had a medical history with the diseases addressed in the stimuli to avoid bias in their responses. Individuals living with a stigmatized disease are more cautious to disclose the information, especially if the disease is not immediately apparent [33,54]. The question arises to what extent the behavior of stigmatized individuals can be simulated under experimental conditions. To further strengthen the validity and generalizability of our results, a follow-up study should examine the perspective of already affected individuals.
Moreover, this was a survey study with limited immersion despite the use of an interactive click dummy. In a follow-up study, researchers could collaborate with health insurers to gather real-world data on upload behavior with a real EHR and an integrated transparency feature as used for this study. Conversely, our study faced limitations due to uncontrolled conditions like participant's location and potential distractions, as participants completed the questionnaire online. Future research could validate our findings through a laboratory study, ensuring a more controlled environment.
Another limitation is that the distribution of our sample in terms of gender, age, and level of education does not correspond to that of the average German population [55,56]. In particular, the level of education of our sample was above average. Although we were unable to detect any effects of the control variables gender, and level of education in the analysis, the results of this study should be validated with a more representative sample in the future."

20) Trial limitations, addressing sources of potential bias, imprecision, and, if relevant, multiplicity of analyses

Sie bearbeiten gerade Ihre Antwort. Wenn Sie die URL freigeben, können andere Personen Ihre Antwort ebenfalls bearbeiten. NEUE ANTWORT AUSFÜLLEN

20-i) Typical limitations in ehealth trials

Typical limitations in ehealth trials: Participants in ehealth trials are rarely blinded. Ehealth trials often look at a multiplicity of outcomes, increasing risk for a Type I error. Discuss biases due to non-use of the intervention/usability issues, biases through informed consent procedures, unexpected events.

	1	2	3	4	5	
subitem not at all important	○	○	○	○	◉	essential

Auswahl löschen

Sie bearbeiten gerade Ihre Antwort. Wenn Sie die URL freigeben, können andere Personen Ihre Antwort ebenfalls bearbeiten.

NEUE ANTWORT AUSFÜLLEN

Does your paper address subitem 20-i? *

Copy and paste relevant sections from the manuscript (include quotes in quotation marks "like this" to indicate direct quotes from your manuscript), or elaborate on this item by providing additional information not in the ms, or briefly explain why the item is not applicable/relevant for your study

"While our manipulation checks for perceived risk (related to stigma) were successful, the manipulation of perceived benefit (related to time course) was not. This may be due to the between-subjects design of our study. In a previous within-subjects design, where participants evaluated both acute and chronic reports, the time course significantly impacted upload behavior [12]. It seems that participants, when comparing multiple conditions, can better discern when uploading is more or less beneficial. In our study, however, participants may have perceived the benefits of uploading as uniformly high, regardless of time course, leading to a diminished ability to detect differences.
It is clear that the adoption and approval of data-gathering technologies are strongly influenced by cultural differences [52]. In comparison to other European nations, the German population exhibits a heightened level of caution regarding the use of personal information online [53]. Given that in this study data collection was conducted solely with residents of Germany, future studies should validate the applicability of these findings in other countries.
We deliberately excluded participants who already had a medical history with the diseases addressed in the stimuli to avoid bias in their responses. Individuals living with a stigmatized disease are more cautious to disclose the information, especially if the disease is not immediately apparent [33,54]. The question arises to what extent the behavior of stigmatized individuals can be simulated under experimental conditions. To further strengthen the validity and generalizability of our results, a follow-up study should examine the perspective of already affected individuals.
Moreover, this was a survey study with limited immersion despite the use of an interactive click dummy. In a follow-up study, researchers could collaborate with health insurers to gather real-world data on upload behavior with a real EHR and an integrated transparency feature as used for this study. Conversely, our study faced limitations due to uncontrolled conditions like participant's location and potential distractions, as participants completed the questionnaire online. Future research could validate our findings through a laboratory study, ensuring a more controlled environment.
Another limitation is that the distribution of our sample in terms of gender, age, and level of education does not correspond to that of the average German population [55,56]. In particular, the level of education of our sample was above average. Although we were unable to detect any effects of the control variables gender, and level of education in the analysis, the results of this study should be validated with a more representative sample in the future."

21) Generalisability (external validity, applicability) of the trial findings

Sie bearbeiten gerade Ihre Antwort. Wenn Sie die URL freigeben, können andere Personen Ihre Antwort ebenfalls bearbeiten.

NEUE ANTWORT AUSFÜLLEN

21-i) Generalizability to other populations

Generalizability to other populations: In particular, discuss generalizability to a general Internet population, outside of a RCT setting, and general patient population, including applicability of the study results for other organizations

	1	2	3	4	5	
subitem not at all important	○	○	○	○	◉	essential

Auswahl löschen

Sie bearbeiten gerade Ihre Antwort. Wenn Sie die URL freigeben, können andere Personen Ihre Antwort ebenfalls bearbeiten.

NEUE ANTWORT AUSFÜLLEN

Does your paper address subitem 21-i?

Copy and paste relevant sections from the manuscript (include quotes in quotation marks "like this" to indicate direct quotes from your manuscript), or elaborate on this item by providing additional information not in the ms, or briefly explain why the item is not applicable/relevant for your study

"While our manipulation checks for perceived risk (related to stigma) were successful, the manipulation of perceived benefit (related to time course) was not. This may be due to the between-subjects design of our study. In a previous within-subjects design, where participants evaluated both acute and chronic reports, the time course significantly impacted upload behavior [12]. It seems that participants, when comparing multiple conditions, can better discern when uploading is more or less beneficial. In our study, however, participants may have perceived the benefits of uploading as uniformly high, regardless of time course, leading to a diminished ability to detect differences.
It is clear that the adoption and approval of data-gathering technologies are strongly influenced by cultural differences [52]. In comparison to other European nations, the German population exhibits a heightened level of caution regarding the use of personal information online [53]. Given that in this study data collection was conducted solely with residents of Germany, future studies should validate the applicability of these findings in other countries.
We deliberately excluded participants who already had a medical history with the diseases addressed in the stimuli to avoid bias in their responses. Individuals living with a stigmatized disease are more cautious to disclose the information, especially if the disease is not immediately apparent [33,54]. The question arises to what extent the behavior of stigmatized individuals can be simulated under experimental conditions. To further strengthen the validity and generalizability of our results, a follow-up study should examine the perspective of already affected individuals.
Moreover, this was a survey study with limited immersion despite the use of an interactive click dummy. In a follow-up study, researchers could collaborate with health insurers to gather real-world data on upload behavior with a real EHR and an integrated transparency feature as used for this study. Conversely, our study faced limitations due to uncontrolled conditions like participant's location and potential distractions, as participants completed the questionnaire online. Future research could validate our findings through a laboratory study, ensuring a more controlled environment.
Another limitation is that the distribution of our sample in terms of gender, age, and level of education does not correspond to that of the average German population [55,56]. In particular, the level of education of our sample was above average. Although we were unable to detect any effects of the control variables gender, and level of education in the analysis, the results of this study should be validated with a more representative sample in the future."

Sie bearbeiten gerade Ihre Antwort. Wenn Sie die URL freigeben, können andere Personen Ihre Antwort ebenfalls bearbeiten.

NEUE ANTWORT AUSFÜLLEN

21-ii) Discuss if there were elements in the RCT that would be different in a routine application setting

Discuss if there were elements in the RCT that would be different in a routine application setting (e.g., prompts/reminders, more human involvement, training sessions or other co-interventions) and what impact the omission of these elements could have on use, adoption, or outcomes if the intervention is applied outside of a RCT setting.

	1	2	3	4	5	
subitem not at all important	○	○	○	○	◉	essential

Auswahl löschen

Does your paper address subitem 21-ii?

Copy and paste relevant sections from the manuscript (include quotes in quotation marks "like this" to indicate direct quotes from your manuscript), or elaborate on this item by providing additional information not in the ms, or briefly explain why the item is not applicable/relevant for your study

Only healthy participants took part in our study. Furthermore, only a theoretical, abstract connection was possible, since the participants did not interact with a real system, rather an clickdummy. In an actual interaction, individual experiences and the personal perception of own personal data may influence behavior.

OTHER INFORMATION

23) Registration number and name of trial registry

Sie bearbeiten gerade Ihre Antwort. Wenn Sie die URL freigeben, können andere Personen Ihre Antwort ebenfalls bearbeiten.

NEUE ANTWORT AUSFÜLLEN

Does your paper address CONSORT subitem 23? *

Copy and paste relevant sections from the manuscript (include quotes in quotation marks "like this" to indicate direct quotes from your manuscript), or elaborate on this item by providing additional information not in the ms, or briefly explain why the item is not applicable/relevant for your study

Deutsches Register Klinischer Studien DRKS00033652

24) Where the full trial protocol can be accessed, if available

Does your paper address CONSORT subitem 24? *

Cite a Multimedia Appendix, other reference, or copy and paste relevant sections from the manuscript (include quotes in quotation marks "like this" to indicate direct quotes from your manuscript), or elaborate on this item by providing additional information not in the ms, or briefly explain why the item is not applicable/relevant for your study

Upon request to the authors

25) Sources of funding and other support (such as supply of drugs), role of funders

Does your paper address CONSORT subitem 25? *

Copy and paste relevant sections from the manuscript (include quotes in quotation marks "like this" to indicate direct quotes from your manuscript), or elaborate on this item by providing additional information not in the ms, or briefly explain why the item is not applicable/relevant for your study

The project was funded by the home institution of the authors. No external funding was required.

X27) Conflicts of Interest (not a CONSORT item)

Sie bearbeiten gerade Ihre Antwort. Wenn Sie die URL freigeben, können andere Personen Ihre Antwort ebenfalls bearbeiten.

NEUE ANTWORT AUSFÜLLEN

X27-i) State the relation of the study team towards the system being evaluated

In addition to the usual declaration of interests (financial or otherwise), also state the relation of the study team towards the system being evaluated, i.e., state if the authors/ evaluators are distinct from or identical with the developers/sponsors of the intervention.

	1	2	3	4	5	
subitem not at all important	○	○	○	○	◉	essential

Auswahl löschen

Does your paper address subitem X27-i?

Copy and paste relevant sections from the manuscript (include quotes in quotation marks "like this" to indicate direct quotes from your manuscript), or elaborate on this item by providing additional information not in the ms, or briefly explain why the item is not applicable/relevant for your study

Since we designed the PFSs. the EHR clickdummy as well as the medical findings ourselves, specifically for this study without any ongoing contributions, there are no relations or conflicts of interest to declare.

About the CONSORT EHEALTH checklist

As a result of using this checklist, did you make changes in your manuscript? *

- ○ yes, major changes
- ○ yes, minor changes
- ◉ no

Sie bearbeiten gerade Ihre Antwort. Wenn Sie die URL freigeben, können andere Personen Ihre Antwort ebenfalls bearbeiten.

NEUE ANTWORT AUSFÜLLEN

What were the most important changes you made as a result of using this checklist?

Not applicable, since we didn't have to make any changes.

How much time did you spend on going through the checklist INCLUDING making changes in your manuscript *

at least 2 hours for answering the questions

As a result of using this checklist, do you think your manuscript has improved? *

- (x) yes
- ( ) no
- ( ) Sonstiges:

Would you like to become involved in the CONSORT EHEALTH group?

This would involve for example becoming involved in participating in a workshop and writing an "Explanation and Elaboration" document

- ( ) yes
- (x) no
- ( ) Sonstiges:

Auswahl löschen

Sie bearbeiten gerade Ihre Antwort. Wenn Sie die URL freigeben, können andere Personen Ihre Antwort ebenfalls bearbeiten.

NEUE ANTWORT AUSFÜLLEN

Any other comments or questions on CONSORT EHEALTH

Meine Antwort

STOP - Save this form as PDF before you click submit
To generate a record that you filled in this form, we recommend to generate a PDF of this page (on a Mac, simply select "print" and then select "print as PDF") before you submit it.

When you submit your (revised) paper to JMIR, please upload the PDF as supplementary file.

Don't worry if some text in the textboxes is cut off, as we still have the complete information in our database. Thank you!

Final step: Click submit !
Click submit so we have your answers in our database!

Senden

Geben Sie niemals Passwörter über Google Formulare weiter.

Dieser Inhalt wurde nicht von Google erstellt und wird von Google auch nicht unterstützt. - Nutzungsbedingungen - Datenschutzerklärung

Does this form look suspicious? Bericht

Google Formulare

Sie bearbeiten gerade Ihre Antwort. Wenn Sie die URL freigeben, können andere Personen Ihre Antwort ebenfalls bearbeiten. NEUE ANTWORT AUSFÜLLEN
